# Deep Learning in Selected Cancers’ Image Analysis—A Survey

**DOI:** 10.3390/jimaging6110121

**Published:** 2020-11-10

**Authors:** Taye Girma Debelee, Samuel Rahimeto Kebede, Friedhelm Schwenker, Zemene Matewos Shewarega

**Affiliations:** 1 Artificial Intelligence Center, 40782 Addis Ababa, Ethiopia; samuelrahimeto@dbu.edu.et (S.R.K.); zemene.matewos@aic.et (Z.M.S.); 2 College of Electrical and Mechanical Engineering, Addis Ababa Science and Technology University, 120611 Addis Ababa, Ethiopia; 3 Department of Electrical and Computer Engineering, Debreberhan University, 445 Debre Berhan, Ethiopia; 4 Institute of Neural Information Processing, University of Ulm, 89081 Ulm, Germany; friedhelm.schwenker@uni-ulm.de

**Keywords:** deep learning, medical image analysis, breast cancer, brain tumor, cervical cancer, colon cancer, lung cancer

## Abstract

Deep learning algorithms have become the first choice as an approach to medical image analysis, face recognition, and emotion recognition. In this survey, several deep-learning-based approaches applied to breast cancer, cervical cancer, brain tumor, colon and lung cancers are studied and reviewed. Deep learning has been applied in almost all of the imaging modalities used for cervical and breast cancers and MRIs for the brain tumor. The result of the review process indicated that deep learning methods have achieved state-of-the-art in tumor detection, segmentation, feature extraction and classification. As presented in this paper, the deep learning approaches were used in three different modes that include training from scratch, transfer learning through freezing some layers of the deep learning network and modifying the architecture to reduce the number of parameters existing in the network. Moreover, the application of deep learning to imaging devices for the detection of various cancer cases has been studied by researchers affiliated to academic and medical institutes in economically developed countries; while, the study has not had much attention in Africa despite the dramatic soar of cancer risks in the continent.

## 1. Introduction

Over the last decades, three different approaches have been practiced to deal with medical images. The first is creating awareness among the community for a regular check-up and it was not be practiced among communities. The second approach is using medical imaging technologies for screening and it is witnessed over the last decades. However, the benefits of medical imaging technology depend on the experience of the image interpreting experts or radiologists. Then, applying a computer-aided detection (CAD) approach using machine learning techniques has brought a promising result along with the imaging technologies. Machine learning techniques have evolved rapidly in recent years to solve complex problems.

**Figure 1:**
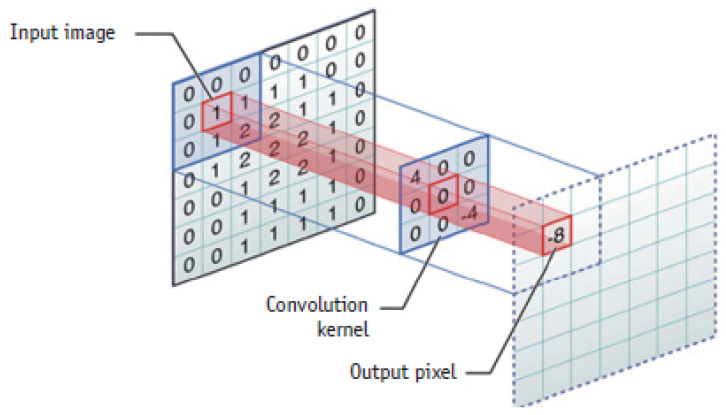
Example of convolution operation from [7].

**Figure 2:**
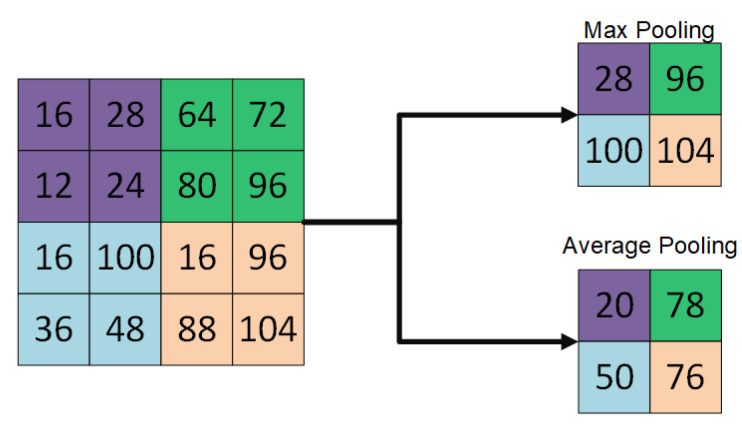
Example of down sampling operation using max-pooling and average-pooling.

The architecture of deep convolutional neural networks (DCNNs) is composed of convolutional layers, pooling layers and fully connected layers to perform feature extraction (see Figure 1), features down sampling (see Figure 2) and classification, respectively during the process of optimization [1].

In convolutional layers, local features such as colors, end-points, corners and oriented-edges are collected in the shallow layers. These local features in the shallow layers are integrated into larger structural features like circles, ellipses, specific shapes or patterns when the layer goes deeper. Afterwards, these features of structures or patterns constitute the high-level semantic representations that describe feature abstraction for each category. In pooling layers, feature down sampling is performed either using average pooling or max-pooling layers to reduce the dimensionality of the features extracted using convolutional layers [2]. On the other hand, in fully connected layers, it takes the extracted features from the convolutional layers as inputs and works as a classifier, well known as multilayer perceptron (MLP). These fully connected layers encode the spatial correspondences of those semantic features and convey the co-occurrence properties between patterns or objects.

There have been many survey papers produced on the application of deep learning on medical image analysis and few among many produced in 2017 are considered in this survey paper. Suzuki [3] in his survey paper claimed that machine learning in deep learning form has emerged in computer vision and paved the way for many researchers to work on medical image analysis using deep learning approach. The popularity of deep learning started after the AlexNet model won the competition in 2012. Suzuki has produced an interesting survey paper that aimed to address four major points: the machine learning techniques used in the computer vision field, changes observed in machine learning after the introduction of deep learning, available machine learning models in deep learning and the impact of deep learning for medical image analysis. As claimed by Litjens et al. [4], convolutional neural network-based deep learning has become a method for medical image analysis. In their survey paper, they considered papers that were related to medical image analysis, specifically for image classification, object detection, segmentation, registration and other tasks. In addition, the areas of application of deep learning were neuro, retinal, pulmonary, digital pathology, breast, cardiac, abdominal and musculoskeletal.

Dinggang Shen et al. [5] claimed that deep learning has helped many researchers in the area of computer vision to identify, classify and quantify patterns in medical images. They specifically argued that deep learning is useful in exploiting hierarchical features from data itself than feature engineering using handcrafting using human effort. Suzuki [6] in his survey paper overviewed the area of deep learning and its application in medical imaging analysis to assess what was changed before and after the introduction of deep learning in machine learning, identifying the reasons that make deep learning powerful and their applications to medical image analysis.

In this survey paper, we briefly describe the breast cancer, cervical cancer, brain tumor, colon cancer and lung cancer along with their respective screening methods. Finally, we reviewed the application of deep learning for each cancer type in terms of deep learning application types like feature extraction, detection, segmentation, prediction and classification. The motivation behind selecting the cancer type for the survey was based on the cancer statistics reported in 2018 by the World Health Organization as presented in Table 1.

## 2. Methods

**Figure 3:**
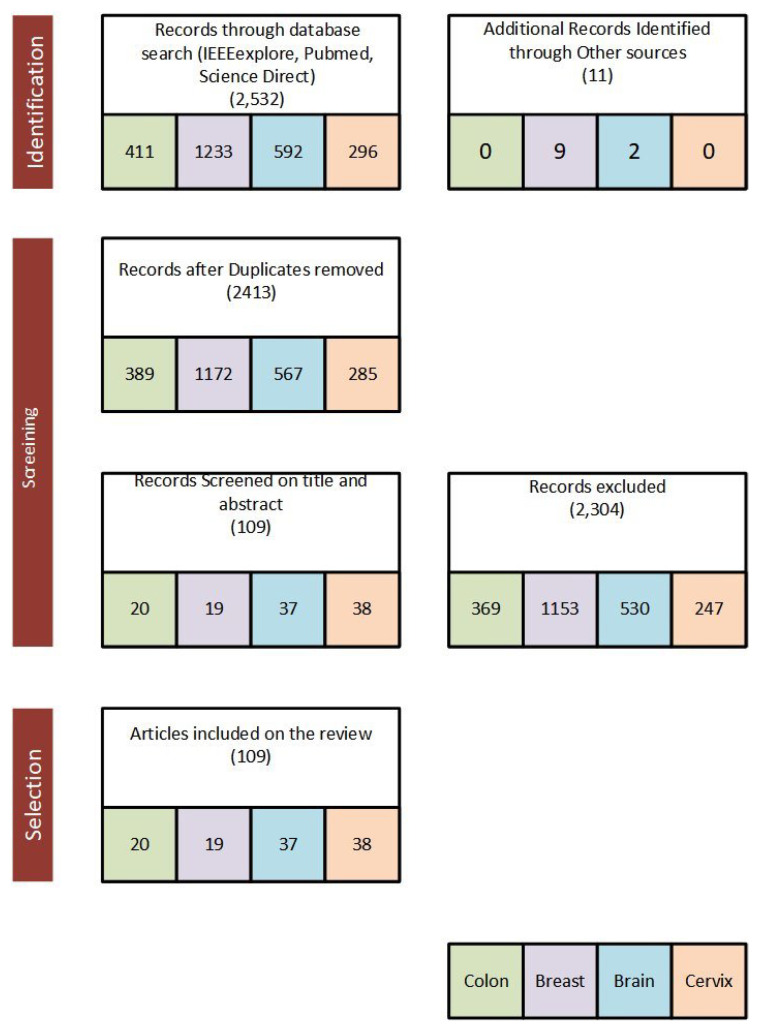
Search criteria framework used for IEEEexplore, PubMed, Google Scholar and Science Direct engines to select papers for review.

Published papers from 2016 to 2020 were considered and reviewed to (1) assess the application of deep learning for breast cancer, (2) assess the application of deep learning for cervical cancer, (3) assess the application of deep learning for a brain tumor and (4) assess the application of deep learning for colon cancer. We first defined a search criterion for the selected search databases. Our general search criteria for this survey paper are ((“colon” OR “colorectal”) AND (“cancer” OR “polyp”) AND (“deep learning”) AND (“Image”) AND (“detection” OR “classification” OR “segmentation” OR “Localization”)) OR ((“breast”) AND (“cancer” OR “mass”) AND (“deep learning”) AND (“Image”) AND (“detection” OR “classification” OR “segmentation” OR “Localization”)) OR ((“Brain”) AND (“Tumor”) AND (“deep learning”)AND (“MRI”) AND (“detection” OR “classification” OR “segmentation” OR “Localization”)) OR (“Cervix” OR “Cervical”) AND (“Deep Learning”) AND (“Classification” OR “segmentation”). The searches were carried out from four databases: (1) PubMed, (2) Science Direct, (3) IEEE Xplore Digital Library and (4) Google Scholar. The search framework of the survey paper is presented in Figure 3 and the major performance metrics used to evaluate deep learning approach applied to the selected medical images are presented in Section 2.1.

### 2.1. Segmentation and Classification Performance Metrics

Most of the performance metrics encountered in the review include area under curve (AUC), sensitivity (Sn), specificity (Sp), accuracy (Acc), precision (P), recall (R), positive predictive values (PPV), Matthews correlation coefficient (MCC), geometric mean (G-Mean), which are usually successful in describing the classification performance [8,9]. Performance measures including Dice similarity coefficient (DSC) or Zijdenbos similarity index (ZSI) or F1-score, Hausdorff distance (H) and intersection over union (IoU) are the most effective metrics for measuring system’s segmentation performance [10]. Here, the true positives for the segmentation are the correctly labeled pixel while it is correctly labeled class for classification case.

## 3. Deep Learning in Tumor Detection, Segmentation and Classification

Region-based segmentation technique was in use in medical image analysis until the deep learning approach evolved in the field of computer vision [8]. However, Lee et al. [7] in their survey paper indicated that the existence of deep learning in the research community has become a reason to use object recognition in an image. In addition to object detection, deep learning has been applied for feature extraction, abnormality detection, cancer/tumor segmentation and classification [11].

### 3.1. Breast Cancer

Breast cancer occurs when there is uncontrolled growth of cells in the breast [12]. It is the most widely diagnosed type of cancer in women and the first prevalent cancer type in Ethiopia [11,13]. There are four types of breast cancer manifestation that include: mass, calcification, architectural distortion and bilateral asymmetry [11].

#### 3.1.1. Screening Methods

As presented in Debelee et al. [11], breast cancer image analysis and breast abnormality detection start with breast cancer screening. Breast cancer screening methods include screen film mammography (SFM), digital mammography (DM), ultrasound (US), magnetic resonance imaging (MRI), digital breast tomosynthesis (DBT) and combinations of the screening methods.

#### 3.1.2. Datasets

There are many datasets prepared for medical image analysis based on the different imaging modalities. The most common and available dataset for the breast cancer is of mammography and histopathology datasets. Some of the most common datasets are discussed in Table 2.

#### 3.1.3. Deep Learning for Detection of Breast Cancer Through Diagnostic Medical Imaging Techniques

Li Shen et al. [2] proposed a deep learning-based breast cancer detection algorithm using end-to-end training approach using mammographic images from the Digital Database for Screening Mammography (DDSM) and INbreast databases. The deep learning architectures used in their paper were ResNet-50 and VGGNet-16. The proposed approach was evaluated in terms of AUC at single model and four-model (ResNet-ResNet, ResNet-VGGNet, VGGNet-VGGNet and VGGNet-ResNet) averaging level. For the DDSM dataset, the best single model achieved a per-image AUC of 0.88, and four-model averaging improved the AUC to 0.91 with sensitivity of 86.1% and specificity of 80.1%. For INbreast database, the best single model achieved per-image AUC of 0.95, and four-model averaging achieved a better AUC value of 0.98 with sensitivity of 86.7% and specificity of 96.1%.

Wu et al. [20] proposed a DCNN architecture based on four columns of ResNet-22 to classify breast cancer screening exams using mammography. There was a total of 200,000 exams which incorporated over 1,000,000 images to train and evaluate the proposed DCNN model. The performance of their network achieved an AUC of 0.895 in predicting whether there is a cancer in the breast, when tested on the screening population and the result was compared to 14 radiologists reading results.

Alzubaidi et al. [21] transfer learning approach on their proposed 74 layer CNN. Their model was pre-trained on one same domain image dataset (erythrocytesIDB dataset, which has images of peripheral blood smears samples taken from patients with Sickle Cell Disease). They divided the original microscopy image into 12 patches and used majority voting for the classification, where the most frequent patch label is chosen to be the image label. The model achieved a patch level accuracy of 90.5% and image-level accuracy of 97.4%. The majority voting they employed seemed not a correct way since if the cells are the majority of normals and if it still has cancerous cells, the system might classify them as normal, which is not good.

Zhu et al. [22] proposed two deep learning approach to predict the occurrence of invasive cancer on MRI images. The first approach was based on transfer learning using GoogleNet pre-trained model to predict the presence of invasive cancer. As a second approach, the authors extracted features from the natural images and used SVM to predict the invasive disease. The best classification result gained in terms of AUC was 0.53 for transfer learning approach and 0.70 for extracted features.

Li et al. [23] explored the abilities of digital breast tomosynthesis (DBT) and full-field digital mammography (FFDM) in mass classification using deep neural networks equipped with or without transfer learning. They also explored an eligible combination strategy of DBT and FFDM in enhancing classification performance. They applied a 16-layer VGG network (VGG-16) and 11-layer deep convolutional neural network (DCNN) for the 2D images and extend the 11-layer DCNN to accommodate the extra dimension in 3D DBT images. The best performer from these methods, a 2D-DCNN which was trained by combining the DBT and FFDM, achieved the highest performance with average AUC, accuracy, sensitivity and specificity of 0.95, 92.13%, 83% and 93.84%, respectively on three class classification (benign, malignant, normal).

Zeiser et al. [24] explored the application of the U-Net model with different depths with or without data augmentation for the segmentation of masses on mammograms. The U-Net model trained with depth of 5 and with data augmentation was the best performer with sensitivity of 92.32%, specificity of 80.47%, accuracy of 85.95%, Dice index of 79.39% and AUC of 86.40% on the DDSM dataset.

Shen et al. [2] applied an ensemble of four best performing deep learning models which were designed based on Resnet50 and VGG16 as patch classifiers and Resnet and VGG blocks as top layer for breast cancer classification. The ensemble of these classifiers achieved the best AUC of 0.91 (sensitivity: 86.1%, specificity: 80.1%) on the detection of benign and malignant masses and classifications on the DDSM dataset.

Zhang et al. [25] used U-net architecture for the segmentation and extraction of fat tissue, fibroglandular tissue (FGT) inside the breast, and all nonbreast tissues outside the breast in breast MRI. They achieved mean DSC of 0.95 for breast and 0.91 for FGT; and mean accuracy of 0.98 for breast and 0.97 for FGT.

Zhou et al. [26] applied 3D deep convolutional neural network (CNN) based on 3D DenseNet [27] architecture with 37 layers for diagnosing breast cancer and localizing the lesions at dynamic contrast enhanced (DCE) MRI data in a weakly supervised manner. The proposed algorithm performance for breast cancer diagnosis showed 83.7% accuracy, 90.8% sensitivity, 69.3% specificity, 0.859 AUC and 0.501 Dice distance.

Summary of the performance of the above reviewed work can be summarized in Table 3.

#### 3.1.4. Deep Learning for Breast Histopathology Image Analysis

Breast histopathology helps to confirm the presence of cancerous sales detected by other imaging modalities. Since histology slides may contain millions of cells and identifying the cancerous sales from the slides is time consuming and tedious job. Hence there are many varieties of research done in this area.

Sheikh et al. [29] proposed a multi-scale input and multi-feature CNN network for the classification of histopathological images. They concatenate four scales (1 ×, 0.5 ×, 0.33 × and 0.25 ×) of the original normalized image to accommodate the scale variant property of the cells and used it as an input to the CNN network. They trained their proposed model on ICIAR2018 and BreakHits datasets. The model achieved a satisfactory max accuracy of 0.83 for the ICIAR2018 dataset and 0.98 for the BreakHis dataset for binary classification. For the multiclass classification, the proposed model’s accuracy was rather unsatisfactory reaching as low as 60% for the ICIAR2018 dataset.

Li et al. [30] modified the Densenet-121 architecture by removing the pooling layers of the 4th Dense-block and feeding the extracted feature maps from each Dense-block to the squeeze-and-excitation (SENet) module for breast histopathology images. The used SENet for receiving more channel-wise information. After concatenating each SENet output, they used a fully-connected layer for the classification purpose. They used a pre-trained Densenet model for their architecture using the transfer-learning approach. Using the publicly available BreakHis dataset, their algorithm achieved an average accuracy of 88% over different magnification levels for binary classification.

Yan et al. [31] used the transfer-learning approach by using Google’s Inception-V3 model as patch-wise feature extraction and image-wise long short-term memory (LSTM) for classifying breast histopathological images into four classes, namely normal, benign, in situ and invasive. They fine-tuned the Inception-V3 model. Their proposed model achieved an average accuracy of 91% on the ICIAR2018 dataset.

Sharma et al. [32] studied the use of pre-trained deep learning networks as feature extractor from breast cancer histopathology images. They used transfer learning on the pre-existing networks (VGG16, VGG19 and ResNet50) for using them as feature extractor. The extracted features were then classified using SVM classifier. The VGG16 network with linear SVM achieved the highest accuracy (93.97% for 40 ×, 92.92% for 100 ×, 91.23% for 200 × and 91.79% for 400 × magnifications).

Vang et al. [33] proposed ensemble classifier and reinforcement backed deep learning approach using inception-V3 for multiclass (normal, benign, in situ and invasive) classification. The ensemble fusion approach for image level prediction involved majority voting, gradient boosting machine (GBM) and logistic regression. Their approach performed low in terms of sensitivity for the two classes (benign and normal). The sensitivity of the normal and benign predicted classes was improved by adding a dual path network (DPN) to use it as feature extractor. However, the extracted features were further sent to the next layer of ensemble prediction fusion using GBM, logistic regression and support vector machine (SVM) to refine predictions. This approach was evaluated in terms of accuracy and scored an accuracy of 87.5%.

Alzubaidi et al. [21] transfer learning approach onto their proposed 74 layer CNN. They pre-trained their model on one same domain image dataset (erythrocytesIDB dataset, which has images of peripheral blood smears samples taken from patients with Sickle Cell Disease). They divided the original microscopy image into 12 patches and used majority voting for the classification, where the most frequent patch label is chosen to be the image label. The model training was don on the ICIAR 2018 dataset. The model achieved patch level accuracy of 90.5% and image level accuracy of 97.4%. The majority voting they employed seemed not a correct way since if the cells are majority of normal and if it still has cancerous cells the system might classify them as normal, which is not good.

The summary of papers explored in classification of breast histological slides can be summarized in Table 4. From the literature reviews the algorithm proposed by Yan et al. [31] seems the best method for histopathological breast cancer detection.

#### 3.1.5. Summary

As presented in Table 5 and Table 6, the deep learning architecture involved in the recently published, from 2016 to 2020, breast cancer we considered in this survey paper were ResNet, VGGNet, AlexNet, Inception V3, U-Net and DenseNet.

As indicated in Table 7, the result of almost all papers in this survey paper were not compared with the domain specialists and performance of the traditional machine learning algorithms.

### 3.2. Cervical Cancer

Cervical cancer is one of the most common cancers among women worldwide, especially in developing nations, and it has a relatively high incidence and mortality rates [36]. Cervical cancer usually develops slowly over time. When cervical cancer begins in the cervix, cervical cells go through changes called dysplasia, in which cells that are not normal begin to appear in the cervical tissue. In its later stage, cancer cells start to multiply and proliferate more deeply into the cervix and to surrounding areas. Fortunately, cervical cancer is mostly preventable with active screening and detection techniques. For example, preventive screening and early detection can decrease the morbidity of cervical cancer by about 70% in the United States [37].

#### 3.2.1. Screening Methods

Nowadays, there are a few frequently-used cervical cancer screening techniques, such as high-risk human papillomavirus (HPV) testing, Pap smear cytology testing, colposcopy and visual inspection of the cervix with acetic acid (VIA), each of which has its advantages and disadvantages.

- **Bimanual pelvic examination.** This is a visual and physical inspection by the physician. It consists of both visual inspections using a device called a speculum and physical inspection by using fingers. This test is not enough on its own and the Pap test is usually performed next.
- **Cervical cytopathology** Papanicolaou Smear (Pap smear) or liquid-based cytology is a process of gently scraping cervical cells and inspection of those cells under a microscope. It can also be analyzed digitally using computers.
- **HPV typing test.** Cervical cancer usually occurs from persistent infection of the cervix with some carcinogenic types of human papillomavirus (HPV) such as HPV16 and HPV18 [38]. It is usually performed along with a Pap test or after Pap test results show abnormal changes to the cervix. The occurrence of HPV does not confirm cancer.
- **Colposcopy.** Colposcopy is a visual inspection of the cervix using a special instrument called a colposcope. The device magnifies the cervix area under inspection like a microscope. It can be used for pregnant women.

Other types of tests were also used for cervical cancer screening such as X-ray, CT scan, MRI and PET scan but they are more expensive and used to detect advanced stages of cancer.

Cervical cytology (Pap test) is the most common test used to look for early changes in cells that can lead to cervical cancer [39]. It has been widely used for the screening of cervical cancer in developed countries and is effective in reducing the number of deaths. It is still unavailable for population-wide screening in the developing countries. This is because screening using cervical cytology is difficult, tedious, time-consuming, expensive and subjected to errors because each slide contains around three million cells with large shape and appearance variation between cells, the poor contrast of cytoplasm boundaries and the overlap between cells [40]. In developed countries like the United Kingdom, cervical cancer screening is performed every 3 years for women aged 25 to 49 years and every 5 years aged 50 to 64 years [41]. Over the past few decades, many types of research were performed in developing a computer-assisted cervical cancer screening method. Most of these researches tried to automatically identify the various stages of cancer or abnormality types by classifying cells on the Pap-smear slides. Most of these classifications consist of cell or nuclei segmentation, feature extraction and classification steps [42].

#### 3.2.2. Datasets for Cervical Cancer

Most of the research regarding the detection and segmentation of cervical cancer used the Herlev dataset. The pap-smear benchmark database provides data for comparing classification methods. The data consists of 917 images of pap-smear cells, classified carefully by cyto-technicians and doctors [43]. The dataset is distributed unevenly into seven classes, namely superficial squamous, squamous intermediate, columnar, moderate dysplasia, moderate dysplasia, severe dysplasia and carcinoma in situ. Each image in the Herlev dataset contains only a single cell. Each slide contains many cells and cells might also overlap. Hussien [44] prepared a more realistic dataset for the classification of cervical cells. Summary of the publicly available datasets for classification and segmentation of cervical cells and cervix is given in Table 8.

#### 3.2.3. Deep Learning for Segmentation of Cervical Cells

Traditional cytological criteria for classifying cervical cell abnormalities are based on the changes in the nucleus to cytoplasm ratio, nuclear size, irregularity of nuclear shape and membrane. In normal cells, the cytoplasm appears much larger than the nucleus with the regular shaped nuclei. Therefore numerous works are focusing on the segmentation of cell or cell components (nuclei, cytoplasm) [41]. Deep learning has been applied for the segmentation of cervical cell nuclei and the whole cell itself. The successful segmentation of each cervical cell from the slides gives a better performance for cancerous cells. Nuclei detection is only maybe helpful and is easier than segmentation of the whole cell and may be enough for the detection of cancer or help for the segmentation of the whole cell.

Song et al. [49] tried to improve cervical cell segmentation by using learning-based segmentation when overlapping cells are encountered. They include high-level shape information to guide the segmentation algorithm which is done by the convolutional neural network algorithm. They evaluated their algorithm in nuclei detection and cervical cell segmentation. By using the datasets ISBI 2015 challenge dataset, and SZU dataset they achieved a Dice similarity coefficient (DSC) of 0.95 and 0.89, respectively.

Zhao et al. [50] proposed an algorithm called Progressive Growing of U-net + (PGU-net +) for Automated Cervical Nuclei Segmentation, which tried to modify the original U-net algorithm [51], which augmented the limited medical dataset for use of deep learning. They claimed these augmentations mix the information of different scales that affect each other; hence, it limits the segmentation accuracy of the model. they proposed a progressive growing U-net (PGU-net +) model, which extracts image features at each scale independently and passing residual information with the next scale. They achieved a segmentation accuracy of 0.925 on the Herlev dataset, with precision 0.901 ± 0.13, recall 0.968 ± 0.04 and ZSI of 0.925 ± 0.09.

Sompawong et al. [52] applied a pre-trained Mask R-CNN for cervical cancer nuclei detection, segmentation and classification into normal and abnormal ones. They used liquid-based histological slides obtained from Thammasat University (TU) Hospital and obtained mean average precision (mAP) of 57.8%, the accuracy of 91.7%, the sensitivity of 91.7% and specificity of 91.7% per image. They used DeepPap as a benchmark to evaluate their algorithm. Since DeepPap used the Herlev dataset (containing images of single cells) for training and testing. It needs to be modified and retrained on the TU dataset. They showed the proposed algorithm performs better than the modified DeepPap on the TU dataset. They did not evaluate the Mask R-CNN algorithm on the Herlev dataset.

Liu et al. [53] proposed a cervical nucleus segmentation method in which pixel-level prior information was utilized to provide the supervisory information for the training of a mask regional convolutional neural network (Mask R-CNN). They added a local fully-connected conditional random field (LFCCRF) to refine the segmentation. Using the Herlev Pap smear dataset, the proposed method achieved 0.96 in both precision and recall and 0.95 in the Zijdenbos similarity index.

Liang et al. [42] used a comparison based detection which combines the decision of two CNN architectures. First reference, samples were obtained by using the ResNet50 with Feature Pyramid Network (FPN) architecture from each cell image from the dataset. At the same time features from the whole slide image, which contains many cells, were extracted through ResNet50 with FPN and region proposal network (RPN). They replaced the original parameter classifier from their baseline network, Faster R-CNN with FPN with their comparison classifier. Their proposed algorithm can detect 11 different cell types from the whole slide. The performance of the proposed algorithm achieved mean average precision (mAP) of 26.3% and average recall (AR) of 35.7%. They argue these performance measurements do not reflect how good the algorithm was since the proposed algorithm groups multiple neighboring cells with the same category into one result.

Kurnianingsih et al. [54] used deep learning methods to segment cervical cells and classify them. For the segmentation purpose, transfer learning was applied on Mask R-CNN weights trained using the COCO dataset. The pre-trained model was trained to segment cervical cell area consisting of both nuclei and cytoplasm. In the segmentation phase, when Mask R-CNN is applied to the whole cell, it outperforms the previous segmentation method in precision (0.92 ± 0.06), recall (0.91 ± 0.05) and ZSI (0.91 ± 0.04).

Deep learning-based segmentation into nuclei segmentation and cell segmentation can be summarized (see Table 9 and Table 10).

#### 3.2.4. Deep Learning for Cervical Cell Classification

Zhang et al. [55] tried to directly classify cervical cells—without prior segmentation—based on deep features, using convolutional neural networks (ConvNets). In their algorithm (DeepPap), a pre-trained ConvNets was trained on a cervical cell dataset consisting of adaptively re-sampled image patches coarsely centered on the nuclei. Then they applied aggregation to average the prediction scores of a similar set of image patches. The proposed algorithm achieved classification accuracy (98.3%), area under the curve (AUC) (0.99) values and specificity (98.3%) on the Herlev dataset.

Hyeon et al. [56] proposed a CNN-based pre-trained model, VGGNet-16 for feature extraction, and use different classifiers namely: logistic regression, random forests, AdaBoost and SVM for classification of the pap-test images into normal and abnormal. From these classifiers, the highest scoring one is the SVM classifier with an F1-score of 0.7817 on a dataset collected locally.

LIN et al. [57] applied the transfer learning approach to fine-tune different CNN models (AlexNet, GoogLeNet, ResNet and DenseNet) which were pre-trained on ImageNet dataset [58]. The pre-trained models were fine-tuned on the Herlev cervical dataset with additional cytoplasm and nucleus morphological masks. They achieved classification accuracies of 94.5%, 71.3% and 64.5%, for two-class (abnormal versus normal), four-class (normal, low-grade squamous intraepithelial lesion (LSIL), high-grade squamous intraepithelial lesion (HSIL) and carcinoma-in-situ (CIS) [59]) and seven-class (”World Health Organization classification system”) classification tasks, respectively.

Chen et al. [60] tried to combine features extracted from different types of tests. They proposed a Faster R-CNN, which is based on Faster R-CNN for fusing acetic and iodine images of the cervix. They fuse non-image features extracted from the cervix transformation zone type, pap test, HPV test and age after they non-linearly compression of the fused image features to 29D by using one fully connected (FC) layer. They did not mention which classifier they used for normal–abnormal classification but achieved an accuracy of 87.4% (88.6% sensitivity and 86.1% specificity) on a locally collected dataset.

Kurnianingsih et al. [54] trained a compact VGG network based on their Mask R-CNN based segmentation algorithm. For the classification, a compact VGG Net classifier yields a sensitivity score of more than 96% with a low standard deviation (± 2.8%) for the binary classification problem and yields a higher result of more than 95% with low standard deviation (maximum 4.2%) for the 7-class problem.

Performance comparison of different pre-trained deep learning models on Pap smear classification was done by Promworn et al. [61]. They compared the performance of CNN models namely resnet101, densenet161, AlexNet, vgg19_bn and squeeznet1_1, which are the top five models based on accuracy in ImageNet. The models are retrained on the Herlev dataset. Based on accuracy, densenet161 was the best performer on both binary classification (94.38%) and multiclass classification (68.54). Based on sensitivity, AlexNet and resnet have achieved 100% on binary classification. Whereas densenet161 was the best performer on multiclass classification with 68.18%. Again, based on specificity, densenet161 was superior with values 82.61% for binary and 69.57% for multiclass classification.

Yutao Ma et al. [62] developed a CADx system by using a convolutional neural network (CNN) for feature extraction and support vector machines (SVM) for classifying the optical coherence microscopy (OCM) images into five classes namely normal, ectropion, low-grade and high-grade squamous intraepithelial lesions (LSIL and HSIL) and cancer. They also used HPV test results for the classification in conjunction with features extracted from the OCM images by the CNN. An 88.3 ± 4.9% classification accuracy was achieved for all five classes. In the binary classification task (low-risk (normal, ectropion and LSIL) vs. high-risk (HSIL and cancer)), the CADx method achieved an area under the curve (AUC) value of 0.959 with 86.7 ± 11.4% sensitivity and 93.5 ± 3.8% specificity.

Ahmed et al. [63] proposed transfer learning-based approaches for the classification of cervical cells. They explored six different methods for the classification of cervical cells by combining three pre-trained models as features, shallow CNN, which consisted of only two convolutional layers and two max-pooling layers, VGG-16 Net and CaffeNet as a feature extraction technique and two classifiers, extreme learning machine (ELM) and auto encoder (AE) for the classification purpose. They used the Herlev dataset for training and testing their system. The best performer from these combinations is the CaffeNet+ELM which achieved a binary classification accuracy of 99.7 and 97.2 for the 7 class classification.

Dong et al. [64] used artificially extracted features such as color, texture and morphology along with the Inception-V3 model for the classification of cervical cells. They used features extracted manually since the features extracted from the CNN architecture since the knowledge of cervical cells is lacking there. Nine artificial features were combined with features extracted from the Inception-V3 architecture joined on the fully connected layer and used the Softmax function for the classification. They keep the aspect ratio of the cells when resizing for the Inception-V3 network will harm the morphological features. The proposed algorithm achieved an overall accuracy of 98.2%, the sensitivity of 99.4% and specificity of 96.73% for normal abnormal classification on the Herlev dataset.

Martinez-Mias et al. [65] tried to improve and make it realistic the cervical classification from PAP smears using a cell merger approach. They used CNN for PAP smear image classification, and optimize and integrate the cell fusion approach since most PAP smear slides contain overlapping cells. They used a local PAP smear dataset collected from ten patients and labeled using biopsy results. Hence, it was used as a gold standard. They trained the CaffeNet model using data prepared using a cell merger to reflect the reality of the PAP smear examination. For classifying the cervical cells into four classes the CaffeNet with the cell merger dataset achieved an average accuracy of just 55.6% with the performance as low as just 16.7% for LSIL class. For the normal/abnormal classification, their proposed algorithm achieved an accuracy, sensitivity and specificity of 88.8%, 0.92 and 0.83, respectively. This performance is satisfactory considering the classification was done on overlapping cell regions.

Xiang et al. [66] used YOLOv3 as a cell detector and Inception-V3-based classifier for cervical cell classification into ten classes that could be present on the slide namely, normal cells (NORMAL), atypical squamous cells-undetermined significance (ASC-US), atypical squamous cells-cannot exclude HSIL(ASC-H), low-grade squamous intraepithelial lesion (LSIL), high-grade squamous intraepithelial lesion (HSIL), atypical glandular cells (AGC), adenocarcinoma (ADE), vaginalis trichomoniasis (VAG), monilia (MON) and dysbacteriosis (DYS). The model achieves 97.5% sensitivity (Sens) and 67.8% specificity (Spec) on cervical cell image-level screening.

The cervical cell classification algorithms can be categorized into two categories, binary and multiclass. In the binary classification cervical cells are classified into normal and abnormal cells (see Table 11). Multiclass classification describing the severity of the cancer including the normal ones (see Table 12).

#### 3.2.5. Deep Learning for Cervix Classification

Colposcopic images are also used for cervical cancer detection using deep learning methods. A colposcopy helps to observe the cervix at up to ×10 magnification [67]. Cervical intraepithelial lesions are easily recognized when treated with acetic acid solutions using colposcopy.

Cervix type classification from smartphone camera was tried in [68] using capsule networks which achieves an accuracy of 94%. A more advanced approach called CervixNet [69] which is designed based on a novel hierarchical convolutional mixture of experts (HCME) method achieved an accuracy of 96.77%.

M. Arora et al. [70] used the transfer learning approach from a pre-trained CNN, Inception V3 model [71] by modifying the output layer. The output layer was replaced by a pooling layer and a fully connected layer for the classification of the cervix based on its image. A cervical image dataset from Kaggle was used here, which has three types of cervix based on the location of the transformation layer. The type of cervix will help the physician whether further tests are needed or not. They obtained an average accuracy of just 54.54%.

Guo et al. [72] explored the application of two versions of regions with convolutional neural networks (R-CNN), Mask R-CNN and Mask${}^{X}$ R-CNN, on three different dataset for cervix classification for automatic segmentation of cervix region. Mask R-CNN is effective on datasets with annotations having the exact boundaries. The Mask${}^{X}$ R-CNN can also trained the bounding box annotation. The highest performance was achieved using Mask R-CNN with Dice and IoU of 0.947 and 0.901, respectively. Mask${}^{X}$ R-CNN also achieved a very good performance with Dice and IoU of 0.92 and 0.86 respectively. These colposcopy images suffer from presense of many distractors such as pubic hair, intra-uterine devices (IUDs), the speculum and even parts of human hand. The main problem in cervix classification from cervix photos is the presence of out of focus images [73].

Guo et al. [74] used an ensemble of three deep learning architectures, RetinaNet, Deep SVDD and a customized CNN for the detection of cervix on smarthphone captured images. They achieved an average accuracy and F1-score of 91.6% and 0.890, respectively.

#### 3.2.6. Summary

Screening through a Pap test for cervical cancer can take days for the final analysis to complete since the pathologist needs to go through millions of cells. A deep learning-based system can detect those cells in minutes if it is accurate enough to be trusted. One of the main challenges of deep learning methods is the presence of other types of cells and other materials present in the image and the overlapping between two adjacent cervical cells. To solve these problems, a large and carefully annotated dataset needs to be built for the algorithms to learn. Building this many datasets for medical images is very difficult. The most commonly used dataset for cervical cancer screening is the Herlev dataset as shown in Table 10.

Deep learning has been applied for cervical cancer screening in many of its screening methods. Most of the successful deep learning-based cervical cancer detection methods were based on a dataset using pap smear histology images. Colposcopic images are also taking more attention since they are easy to take and not invasive. Their accuracies on the detection of cervical cancers is not as good as that of the histology images. Cervical cancer detection using the deep learning methods based on the colposcopic images are becoming common since a large dataset can be collected and annotated easily. Colposcopic screening could be applied for mass screening purposes with the aid of deep learning, since taking samples are easy. As we can see from Table 9, Table 10, Table 11 and Table 12, the Herlev dataset is the most used dataset for cervical cell classification and segmentation works. Most of the coloscopic datasets used for cervix classifications are locally collected. We can also see that the deep learning methods for nuclei segmentation are more accurate than that of cell segmentations since cell boundaries might overlap between adjacent cells. For the classification case, binary classifiers are more accurate than that of multiclass classifiers which can detect the type of abnormalities in the cells.

From Table 11, Table 12 and Table 13, we can see those deep learning methods with pre-trained networks and those with transfer learning mechanisms are more accurate than networks trained from scratch with TensorFlow the widely used software.

Most of the reviewed papers on the application of deep learning on cervical cancer screening were published in 2019 with an average impact factor of 3.4 (see Table 14). And, as shown in Table 15, only one of the papers compares the algorithm performance with that of the specialist.

### 3.3. Brain Tumor

Brain tumor is a group of abnormal cells around or inside the brain due to the uncontrolled division of cells with a serious effect of deterring the normal functionality of the brain activity and destroying the healthy cells[75].

Brain tumor is classified into benign or low-grade (grade I and II) and malignant or high-grade (grade III and IV). Benign is a non-cancerous tumor that does not exhibit any progression and cannot spread to other parts of the body; it started in the brain with a very low growth rate. On the other hand, a malignant tumor is cancerous with an attribute of growing rapidly and spreading to other parts of the body. Malignant tumors can further be categorized as primary and secondary. Primary malignant tumor originates in the brain itself; whereas, the secondary type begins from somewhere else in the body and spreads to the brain. Cancerous cells that spread to the brain commonly originate from the lung, kidney, breast, skin and colon. A metastatic brain tumor is another expression for this type of brain tumor. Glioblastoma multiform (GBM) is the most common type of primary brain tumor that grows fast from glial cells. An intense clinical treatment plan is required for high-grade gliomas (HGG) as they have a higher spreading rate than the low-grade gliomas (LCG) [76]. It is evidenced that patients with GBMs decease in less than a year. Early detection helps a therapeutic plan of patients and improves the overall survival rate [77]. The most prevalent brain cancer is high-grade glioma with 85% of new cases of malignant primary tumor diagnosed every year [78].

#### 3.3.1. Screening Methods

Magnetic resonance imaging is the most common brain tumor diagnosis and has a great role in treatment planning strategies [79]. These images have an important contribution towards an automatic medical image analysis field as they provide quite a lot of information about the brain structure and abnormalities [80].

This is the reason why MRI images have a great impact on the automatic medical image analysis field. There are various steps taken in the course of brain tumor treatment. The first step is determining if the tumor does exist in the brain or not. Then, the infected region in the brain tissues must be extracted from an MRI image in a process called segmentation [81]. Segmentation is not an easy task as MRI images may not help human readers easily discern regions of concern for various technical reasons. However, segmentation is a very important task in properly conducting the diagnosis, treatment and appraisal of treatment outcomes. A great number of automatic segmentation methods with varying degrees of accuracy have been developed as applications of the computational science for utilization of imaging devices advance.

There are different modalities of the MRI including T1-weighted (T1), T2-weighted contrast-enhanced (T1c), T2-weighted (T2) and T2-weighted fluid attenuated inversion recovery (FLAIR) for segmenting the brain tumor [82]. Moreover, features of the MRI like textures, local histograms and structure tensor eigenvalues have been used in brain tumor segmentation [83]. Deep learning-based methods have become state-of-the-art as they score superior performance in image analysis fields [84].

#### 3.3.2. Datasets

Most of the researchers have applied publicly available brain tumor image datasets to test their methods. Summary of publicly available datasets for brain tumor segmentation and classification is summarized in Table 16.

#### 3.3.3. Deep Learning in Brain Tumor Segmentation

Alkassar et al. [91] proposed transfer learning and fully convolution network (FCN) to achieve robust tumor segmentation using VGG-16 networks. The proposed method achieved a global accuracy of 0.97785 and a 0.89 Dice score in terms of whole tumor segmentation on MRI images from the BRATS2015 dataset.

Amiri et al. [92] proposed a simple and reliable brain segmentation method in MRI images through recursively and deeply transferring a learned random forest (RF) to guide an SVM classifier for segmenting tumor lesions while capturing the complex characteristics of brain tumor appearance. They tested this method on 20 patients with high-grade gliomas from the Brain Tumor Image Segmentation Challenge (BRATS) dataset. Their method outperforms both SVM and RF with a high statistical significance using paired t-test; i.e., a mean Dice index of 72% compared to SVM (59%) and RF (63%).

Chahal et al. [93] proposed a novel approach using deep learning which utilizes both global and local brain image datasets for precise segmentation. Their proposed deep learning model combines two-pathway and cascade architectures to analyze and implement brain segmentation. The results are evaluated over Input Cascade and the outcomes showed better performance—that is, a metrics of Dice score for high grade and low-grade image with values 0.943 and 0.950, respectively—than existing MFC cascade.

Ding et al. [94] proposed deep residual dilate network with middle supervision (RDM-Net) which combines the residual network with dilated convolution. By evaluating their framework on the BRATS 2015 challenge, their framework proved to achieve better performance than other state-of-the-art methods incomplete tumor (Dice score of 0.86) and core tumor segmentation (Dice score of 0.78). However, the Dice score for enhancing tumors only achieves 0.63 which is not as good as the other counterpart methods. The reason for this could be the focus on the 2D slices segmentation by the proposed framework which pays less attention to the context information within slices by comparing with 3D segmentation. The loss of context information may lead to worse performance on the enhancing tumor segmentation.

Mallick et al. [95] have used a deep wavelet autoencoder (DWA) for an image compression technique which blends the basic feature reduction property of autoencoder along with image decomposition property of wavelet transform for further classification task by using DNN. The performance of the DWA-DNN classifier was compared with other existing classifiers like autoencoder-DNN or DNN and the proposed method surpasses them all with an overall accuracy of 96% where that of AE-DNN is 93% and DNN is 91%.

Ramirez et al. [96] proposed a new variational model for saliency detection in images and its application to brain tumor segmentation. The model works by incorporating a saliency term to a classical total variation-based restoration functional and hence discriminates what is relevant (salient) from the background. They have, therefore, introduced a deep learning framework for using available knowledge from a specific application to optimize the parameters of the energy functional. The proposed framework achieved a Dice score of 0.857, precision 0.845 and recall 0.882.

Sajid et al. [97] proposed a deep learning-based method that uses different modalities of MRI for the segmentation of brain tumors. The proposed hybrid convolutional neural network architecture uses a patch-based approach and deals with the over-fitting problems by utilizing dropout regularize alongside batch normalization, whereas the data imbalance problem is dealt with by using a two-phase training procedure. The proposed method contains a preprocessing step, in which images are normalized and bias field corrected, a feed-forward pass through a CNN and a post-processing step as a means of removing remnant false positives in the skull portion. The proposed method is validated on the BRATS 2013 dataset, where it achieves scores of 0.86, 0.86 and 0.91 in terms of Dice score, sensitivity and specificity for whole tumor region, improving results compared to existing state-of-the-art techniques.

Wang et al. [98] proposed an automatic method named residual and pyramid pool network (WRN-PPNet) to segment brain tumor by first obtaining 2D slices from 3D MRI brain tumor images and then normalizing the 2D slices and putting them in the model. The model will output the tumor segmentation results. The experimental results show that the proposed method is simple and robust compared to the other state-of-the-art methods with an average Dice, sensitivity and PPV values on randomly selected datasets 0.94, 0.92 and 0.97, respectively.

Zhao et al. [99] proposed a new method for brain segmentation which is an integration of fully convolutional neural networks (FCNNs) and conditional random fields (CRFs) in a unified framework. The result helps to obtain segmentation results with the appearance and spatial consistency. The following steps are taken to for training the deep learning model using 2D image patches and image slices: (1) training FCNNs using image patches; (2) training CRFs as recurrent neural networks (CTF-RNN) using image slices with parameters of FCNNs fixed; and (3) fine-tuning the FCNNs and the CRF-RNN using image slices. In the model, 3 segmentation models are particularly trained using 2D image patches and slices obtained in axial, coronal, and sagittal views respectively are combined to segment brain tumors using a voting-based fusion strategy. The method used BRTS 2013, BRATS 2015 and BRATS 2016 with an experimental result of a competitive score. The method achieved a promising performance on the BRATS 2013 and BRATS 2015 testing dataset. The method could also achieve competitive performance with only 3 imaging modalities (FLAIR, T1c and T2) rather than 4(FLAIR, T1, T1c and T2). In BRATS 2016, the method ranked first on its multi-temporal evaluation.

Kuzina et al. [100] proposed a knowledge transfer method between diseases via the generative Bayesian prior network to mitigate the common challenge of obtaining large image datasets for automatic segmentation. They have applied deep weight prior; hence the name UNet-DWP for their method, to incorporate information about the structure of previously learned convolutional filters during the training of a new model. A comparison between a pre-trained approach and random initialization to this approach proves that this method yields the best results in terms of the Dice similarity coefficient metric on a small subset of the BRATS2018 dataset. The performance of the model was rated by taking subsets containing 5, 10, 15 or 20 randomly selected images from the dataset and comparing them with the fixed test sample size of 50 images. They have also used a blend of binary cross-entropy and Dice losses to train U-Net in the non-Bayesian setting. The results indicate that the model outperforms both pre-trained and randomly initialized U-Nets for all the training sizes.

Zeineldin et al. [101] proposed a new generic deep learning architecture named DeepSeg to address the challenge of distinguishing tumor boundaries from healthy cells in the brain tumor diagnosis. This method helps to wholly automate detection and segmentation of the brain lesion using FLAIR MRI data. The developed system is a decoupling framework interacting encoding and decoding relationship where the encoder part performs spatial information using a convolutional neural network and the decoder provides the full-resolution probability map from the resulting semantic map. The study has employed different CNN models such as residual neural network (ResNet), dense convolutional network (DenseNet) and NASNet using modified U-Net architecture. The proposed architecture has been tested on MRI datasets of brain tumor segmentation (BRATS2019) challenge which includes s336 cases as training data and 125 cases for validation data yielding Dice and Hausdorff distance scored of about 0.81 to 0.84 and 9.8 to 19.7, respectively. The proposed DeepSeg is open source and freely available at https://github.com/razeineldin/DeepSeg/.

Fabelo et al. [102] suggested a deep learning-based hyperspectral image (HSI) processing modality to be used as a reliable support in real-time neurosurgical procedure for carrying out accurate resection of the tumor without affecting much of the normal brain tissue. The study employed a number of deep learning techniques for the detection of brain tumors using HSI. The HS image database was obtained during the course of operation and the system employed a highly sophisticated and specialized visible and near-infrared (VNIR) push broom camera. Classification methods with 2D-CNN and pixel-wise classification with 1D-DNN have been found to yield a very good result. Despite the challenge in obtaining sufficient number of training samples and the anomalies incurred due to brain movement during scanning, the overall average accuracy for the proposed method was 80%. The method has also achieved a very high specificity for both binary and multiclass classification schemes with values of 100% and 90%, respectively.

The summary of researches on deep learning methods for brain tumor segmentation is presented in Table 17.

#### 3.3.4. Deep Learning in Brain Tumor Classification

Like in the segmentation, deep learning-based methods have performed fairly well in image classification of brain tumors. Yet, variation in the shape, size, location and contrast of tumor tissue cells is the major factor that impacts the accurate classification of brain tumors from MRI images [103].

Deep learning techniques involving different enhancement methods are used to classify different types of brain tumors—glioma, meningioma and pituitary. The classification is further categorized into axial, coronal and sagittal planes that are used by various algorithms to minimize the error rate of neural networks in identifying the brain tumor [104].

Mohsen et al. [80] employed a DNN classifier where a 7-fold cross-validation technique was applied for building and training the DNN of 7-hidden layers structure for classifying a dataset of brain MRIs into four classes, i.e., normal, glioblastoma, sarcoma and metastatic bronchogenic carcinoma. They have combined the classifier with the discrete wavelet transform (DWT)—a powerful feature extraction tool—and principal components analysis (PCA). They achieved a classification rate of 96.97%, recall 0.97, precision 0.97, F-measure 0.97 and AUC (ROC) 0.984.

Alqudah et al. [105] used a convolutional neural network (CNN) for classifying a dataset of 3064 T1 weighted contrast-enhanced brain MR images for grading the brain tumors into three classes called glioma, meningioma and pituitary. The research has used T1-weighted contrast-enhanced brain MR images for classifying brain tumor grades. They have used a free online available dataset at [90] which contains images having the above-mentioned attributes. A total of 18 layers in the proposed CNN architecture would enable the classifier to rate the brain tumor effectively. In their work they proved that the proposed CNN classifier is a powerful tool with an accuracy of 98.93% and sensitivity 98.18% for cropped lesions; for the uncropped lesions, they have obtained an accuracy of 99% and 98.52% sensitivity; for segmented lesion images, the result is 97.62 accuracy and 97.40% sensitivity.

Ucuzal et al. [106] developed a deep learning free web-based software that can be utilized in the detection and diagnosis of the three types of brain tumors (glioma/meningioma/pituitary) on T1-weighted magnetic resonance imaging. In the research, 3064 T1-weighted MR image scans for the three types of brain tumors have been used. Out of which, 2599 instances were used in the training phase; whereas, the remaining 465 were used in the testing phase. A python programming language library called Auto Keras was used in image pre-processing (image rotation, changing width and length, truncating images, rescaling, etc). Furthermore, a Bayesian optimization technique was used to tune the hyperparameters of the model. With this, they have verified that all the calculated performance metrics—i.e., accuracy, precision, sensitivity, specificity, F1-score, MCC, G-Mean of the experimental results are higher than 98% for classifying the types of brain tumors on the testing dataset obtained from Nanfang Hospital and Tianjin Medical University General Hospital which is an open-source dataset downloaded from [107]. This data set consists of 3064 T1-weighted contrast-enhanced MR images from 233 patients: 708 meningiomas, 1426 glioma and 930 pituitary tumors. The developed web-based software can be publicly available at [108].

Selvy et al. [109] developed a model that makes use of an image processing technique and artificial neural network for successful detection of the brain tumor. To enhance the contrast of the original image in its analysis and manipulation, they have used histogram equalization (HE) technique where gray level co-occurrence matrix (GLCM) would be used on the feature extraction. A probabilistic neural network (PNN) classifier is applied to the obtained feature to accurately determine tumor location in brain MRI images. The PNN classifier has produced an accuracy of 90.9%, specificity of 100% and sensitivity 85.75%.

Sultan et al. [110] proposed a deep learning (DL) model to classify different brain tumor types. The model which bases a convolutional neural network has employed two publicly available datasets acquired from Nanfang Hospital and General Hospital, Tianjing Medical University, China from 2005 to 2010. The two datasets entail 233 and 73 patients with a total of 3064 and 516 images on T1-weighted contrast-enhanced images, respectively. The overall accuracy of the proposed network is 96.13% for the first and 98.7% for the second dataset. The result inferred that the model has the ability to perform brain tumor multi-classification. The network training and performance computations are finally presented. The system parameters used to train the neural network structure are Intel i7-7700HQ CPU (2.8 GHz), NVIDIA GTX 1060 (6 GB) GPU, 16GB RAM, Matlab 2018b and Python 3. The network is constructed from 16 layers where the input layer holds the pre-processed images passing through the convolution layers and their activation functions (3 convolution, 3 ReLU, normalization and 3 max-pooling layers). Additionally, two dropout layers are used to prevent overfitting followed by a fully connected layer and a softmax layer to predict the output and finally a classification layer that produces the predicted class. Although the dataset is relatively not big (due to the variety of imaging views), data augmentation helped well to show better results and hence, overcome this problem.

Badža and Barjaktarovic [111] presented a new CNN architecture for Brain Tumor Image Segmentation and classification for three tumor types. The study has employed an image database that contains 3064 T1-weighted contrast-enhanced MRI images acquired from Nanfang Hospital and General Hospital, Tianjin Medical University, China from 2005 to 2010. The input layer of the proposed network was represented by MRI images of the database after being normalized to 256 × 256 pixels. The network architecture having consisted of input, two main blocks, classification block and output was employed to perform tumor classification. The blocks consist of the rectified linear unit (ReLU) activation layer, the dropout layer and the max-pooling layer engaged in fine-tuning and resizing the images. A CNN developed in Matlab R2018a (The MathWorks) was employed for the tumor classification. The evaluation of the network was assessed using four approaches: combinations of two 10-fold cross-validation methods and the two databases mentioned above. The generalization capability of the network was tested with one of the 10-fold methods, subject-wise cross-validation, and the improvement was tested by using an augmented image database. The best result for the 10-fold cross-validation method was obtained for the record-wise cross-validation for the augmented data set, and, in that case, the accuracy was 96.56%. With good generalization capability and good execution speed, the new developed CNN architecture could be used as an effective decision-support tool for radiologists in medical diagnostics.

The summary of the papers reviewed is presented in Table 18.

#### 3.3.5. Summary

As shown in Table 19, papers from 2016,2018, 2019, and 2020 were reviewed here. Unlike other cancer type papers, reviewed papers on brain tumors consist of a significant number of papers that compared the performance of their model with that of the domain expert(see Table 20).

As indicated in Table 17 and Table 18, Brain Tumor Image Segmentation Challenge (BRATS) of various versions is the most widely used dataset among the researchers and appeared in ten out of the seventeen papers reviewed.

From Table 21 it was found that a Tensor flow-based framework run by a high-speed core processor or a GPU was widely used, seven out of seventeen, followed by PyTorch, two out of seventeen, to implement the experiments and conducting the deep learning training. However, the rest of the papers have not explicitly indicated which software platform they have applied. On the other hand, VGGNet is the most frequently applied network to achieve robust tumor segmentation 52.9% of the papers have made a comparison with domain experts; on the other hand, 94.1% of the paper has made a comparison with the traditional method.

### 3.4. Colorectal Cancer (CRC)

Worldwide in 2018, more than 1,849,518 (which accounts for 10.2% of overall cancer cases) new cases of colorectal cancer (CRC) are diagnosed and nearly 880,792 people died which is 9.2% of all cancer-related deaths [112]. It is the third most common cancer worldwide and the second most deadly [112]. Since colorectal cancer takes a long time before it becomes invasive, it is often curable if found early. Hence, casual screening for colorectal cancer can substantially reduce its mortality. Approximately 95% of all colorectal cancers are adenocarcinomas [113]. Colorectal adenocarcinomas develop in the lining of the colon or rectum and are characterized by glandular formation.

#### 3.4.1. Screening Methods

There are three common screening methods for colorectal cancer: fecal occult blood test (FOBt), flexible sigmoidoscopy (FS) and total colonoscopy (TC) [114]. FOBt reveals traces of blood in stool samples which is an early sign of colorectal cancer. FS involves visual inspection of the distal bowel for polyps and cancers. TC visualizes the entire bowel and therefore is a more invasive examination. The advancement of whole slide imaging (WSI) scanners has opened new opportunities in automating pathology image analysis by digitizing the slides [115]. Histological examination of the glands, most frequently with the hematoxylin & eosin (H & E) stain, is routine practice for assessing the differentiation of cancer within colorectal adenocarcinoma [113]. Pathologists use the degree of glandular formation as an important factor in deciding the grade of the tumor. Accurate segmentation of structures of the glandular formations such as glands and nuclei have crucial importance, because their morphological properties can assist the pathologist in screening the malignancy [113].

#### 3.4.2. Datasets

In Table 22, we present some of the publicly available and widely used datasets for colorectal cancer detection and segmentation.

#### 3.4.3. Deep Learning for Cell Detection and Classification on Histological Slides

Kainz et al. [120] applied deep learning methods to segment and classify colon glands into benign and malignant types for GlaS@MICCAI2015 challenge. They first pre-processed the stained RGB by taking the Red channel and ignoring the others. Then contrast enhancement was performed using contrast limited adaptive histogram equalization (CLAHE) technique. Two CNN classifiers were trained: Object-Net, Separator-Net. Object-Net is for the detection of benign and malignant glands from their respective backgrounds. Separator-Net is for classifying gland-separating structures since the Object-Net architecture segment two neighboring glands as one. These to classifiers are then regularized using a figure-ground segmentation based on weighted total variation to produce the final segmentation result. They have achieved 96% average accuracy on the two tests provided by the challenge.

Sirinukunwattana et al. [121] proposed a spatially constrained convolutional neural network (SC-CNN) that includes parameter estimation layer and spatially constrained layer for spatial regression to predict the probability of a pixel being the center of a nucleus in hematoxylin and eosin (H & E) stained histopathology images. For classifying the detected nuclei they combine neighboring ensemble predictor (NEP) with a standard softmax CNN (s-CNN). For the nuclei detection using SC-CNN, they achieved 0.77 precision, 0.82 recall, and 0.8 F1-score. The NEP&s-CNN classifier achieved an F1-score of 0.784 and the overall nuclei detection and classification (SC-CNN+NEP&s-CNN) achieved an F1-score of 0.69.

Graham et al. [113] used a fully convolutional neural network that counters the loss of information caused by the max-pooling layer by introducing original down-sampled image into the residual unit using the minimal information loss (MIL) units. They applied atrous spatial pyramid pooling for multi-level aggregation and preserving the resolution. They achieved an F1-score of 0.92 for gland segmentation using the GlaS challenge dataset.

Chamanzar et al. [122] develop a deep learning method that can detect and segment a single cell using only point labeled dataset. They combined Voronoi transformation, Local pixel clustering and Repel encoding methods with U net with Resnet encoder by feeding them to a multi-task scheduler for training the system. They achieved an accuracy of 93% for cell segmentation and 94.1% for detection of adenocarcinoma.

Sari et al. [123] proposed a novel approach for feature extraction, which defines the features by considering only the salient subregions of the image. The salient subregions were detected by the detection of nuclear and non-nuclear pixels using an algorithm presented in [124]. Then a deep belief network of restricted Boltzmann machines (RBMs) re-characterizes these regions and extract features. These features are clustered using the k-means clustering algorithm and SVM classifier for categorizing those regions. They achieved an average precision, recall, and F1-score of 82.3%, 89.9% and 85.1, respectively at the detection of colon adenocarcinoma.

Shapcott et al. [125] proposed a deep learning-based cell identification on histological images of the colon with a systematic random sampling of the WSI slides. Their proposed system consists of two CNNs in series in which the first one detects cells on the WSI slide while the second one classifies those cells into epithelial, inflammatory, a fibroblast or ”other”. The training was performed on a local dataset and Evaluated using the ”The Cancer Genome Atlas (TCGA)” dataset. Using five patients’ slides, they achieved an average accuracy of 65% in the detection of cells and 76% in the classification.

Tang et al. [126] proposed Segnet based gland segmentation on the histology image of the colon. Augmented MICCAI2015 challenge dataset was used to train the SegNet network which is a CNN with encode-decoder architecture for pixel-wise segmentation. SegNet achieved an average Dice similarity index of 0.872 and Hausdorff distance of 104.61.

Vuong et al. [127] proposed an algorithm based on DenseNet121 that can perform both classification and regression tasks on WSI images, for improving the overall performance of the system. They designed this multi-task deep learning model by adding two fully connected layers, one for classification and one for regression, after the DenseNet121 network. The classifier classifies the tissue image into four distinctive pathologies and the regressor considers these four pathological categories as continuous values. They achieved 85.1% accuracy in classifying colon tissues into four categories.

Sabol et al. [128] proposed a semantically explainable fuzzy classifier called cumulative fuzzy class membership criterion (CFCMC) for classifying WSI of colorectal cancer tissue into eight different tissue types. They compared many CNN architectures as feature extraction for the CFCMC classifier with the Xception architecture performance being the best feature extractor for the CFCMC. The explainability of the system is its ability to provide a degree of confidence for each of its predictions. The proposed method achieved an accuracy of 92.78% for the classification of the different tissue samples. The explainability was evaluated by pathologists based on its objectivity, level of details, reliability and quality. Based on these measures, they confirmed that the explainability of the system is better than the traditional CNN architectures.

#### 3.4.4. Deep Learning for Classification of Polyps on Endoscopic Images

Colorectal polyps are abnormalities in the colon tissues that can develop into colorectal cancer. The survival rate for patients is higher when the disease is detected at an early stage and polyps can be removed before they develop into malignant tumors. These tests are usually performed using endoscopic analysis of the colon. During this study, the endoscopist explores the colon cavity looking for abnormal growths of tissue, polyps. However, polyp detection is a challenging problem given its high variation in appearance, size, shape and in many cases its high similarity with the surrounding tissue.

The application of CTs for the screening of colorectal cancer suffers from false positives due to the similarity between polyps and colorectal tubes on the CT image. Approaches in [129] can help to distinguish between colorectal tubes and polyps in CT scans of the colon area using a three dimensional massive-training artificial neural network (3D-MTANN). The proposed model manages to reduce false positives by 33% while keeping a sensitivity of 96%.

Ornela Bardhi et al. [130] used CNNs with auto-encoders for the automatic detection of colon polyp. They used the SegNet architecture from the TensorFlow to build the model and train it from scratch using three datasets: CVC-ColonDB, CVC-ClinicDB and ETIS-LaribPolypDB. They achieved a maximum accuracy of 96.7% on the EITS dataset for the detection of colon polyps.

Bour et al. [131] trained different architectures: ResNet50, ResNet101, Xception, VGG19 and Inception V3 for classification of polyps. ResNet50 achieved the highest accuracy of 87.1 % with precision 87.1%, recall 87.1%, F1-score 87.1% and specificity 93%.

Liu et al. [132] used a deep learning network, faster_rcnn_inception_resnet_v2 model for localization and classification of endoscopic images of the colon. They achieved 90.645% mean average precision and 0.5 for the intersection over union (IoU).

Ozawa et al. [133] used deep convolutional neural network (CNN) architecture called single shot multibox detector (SSD) for the detection of colorectal polyps. All layers were fine-tuned using stochastic gradient descent with a global learning rate of 0.0001. The trained SSD detected the trained CNN detected 1246 colorectal polyps from a dataset collected at Tada Tomohiro Institute of Gastroenterology and Proctology with a sensitivity of 92% and a positive predictive value (PPV) of 86%.

Nadimi et al. [134] used a modified version of ZF-net, a CNN architecture proposed by Matthew D. Zeiler and Rob Fergus [135], as the basis for a Faster R-CNN to localize regions of images containing colorectal polyps. They trained their architectures using a locally collected dataset labeled as colorectal polyps (N = 4800) and normal mucosa (N = 6500). The proposed architecture achieved an accuracy, sensitivity and specificity of 98.0%, 98.1% and 96.3%, respectively. The proposed approach produces the bounding box annotation of the polyp.

#### 3.4.5. Summary

The most common colorectal cancer screening methods use endoscopic images to find abnormal colon tissues, polyps and locating cancerous cells or glands on WSI images. Hence most of the application of deep learning for detecting colorectal cancer is either finding adenocarcinoma on WSI or detection of polyps on colonoscopic images. Most of the research shows promising results in both polyp detection and adenocarcinoma or glands detection as seen in Table 23 and Table 24.

The main challenge for the analysis of colposcopic images is most of the dataset suffers from out of focus problems. Detection of polyps from endoscopic images presents a big opportunity for deep learning methods to shine since most of the physicians may miss smaller polyps. Still, challenges are there due to low-quality samples and the operator might miss some areas.

Papers who used pre-trained models and applied transfer learning approaches discuss their findings in detail and are from reputable journals (see Table 25 and Table 26). From Table 27, only two of the papers measure the performance of their proposed model against expert physicians.

### 3.5. Lung Cancer

Lung cancer is the deadliest cancer worldwide with the highest incidence rate [112]. The death rate is so high because the majority of the cases are discovered at an advanced stage, where curative treatment is no longer feasible. Hence, screening for early detection is significant enough for decreasing mortality.

#### 3.5.1. Screening Methods

The recommended screening test for lung cancer is low-dose computed tomography (LDCT), which uses a low dose of X-ray radiation to get detailed images of the lungs. Physicians will study different slices of the LDCT to determine the presence of an abnormal area called lung nodule (or mass) [136]. Other approaches also exist like chest X-ray(the usual X-ray image), sputum cytology (microscopic analysis of mucus from the lungs). According to a study performed in [136], these approaches do not seem to decrease the mortality rate.

#### 3.5.2. Datasets

In Table 28, some of the publicly available and widely used datasets for lung cancer detection and segmentation are listed.

#### 3.5.3. Deep Learning for Lung Nodules Detection

Before the introduction of convolution-based deep learning methods, Suzuki [140] uses massive training artificial neural networks (MTANN) for the detection and decreasing false positives in lobules detection using extracted subregions from LDCT images. MTANNs are designed based on the concepts of artificial neural network filters, where MTANN will classify each subregion (kernel) independently. Hence, the convolution process is external. They used Multiple MTANNs in parallel to distinguish between nodules and non-nodules by training each MTANN with the same nodules but different types of non-nodules. multi-MTANN was applied to differentiate between benign and malignant nodules from LDCT images in [141].

Tajbakhsh and Suzuki [142] compared the performances of the two widely studied deep learning models, CNNs and MTANNs, for the detection and classification of lung nodules. The proposed MTANN-based architecture outperforms the best performing CNN (AlexNet in their experiment) insensitivity and false-positive rates achieving 100% sensitivity and 2.7 false positives per patient. In the classifications of the nodules into benign and malignant the MTANNs achieved an AUC of 0.88.

Gu et al. [143] proposed a novel CAD system for the detection of lung nodules using a 3D-CNN. They implemented a multiscale approach for making the system more efficient at the detection of various sizes of nodules. The proposed CAD system considers the preprocessing step, which is usually essential in building a standalone CAD system. It has a volume segmentation step for generating ROI cubes for the 3D-CNN to classify them. After the classification, DBSCAN was used to merge neighboring regions into one since they might be different parts of the same nodule. Using the LUNA16 dataset, they found out that small scale cubes are efficient in the detection of smaller nodules (92% sensitivity and four false positives per patient), but larger ones have lower sensitivity (88%), but with an average of one false positive per patient.

Sahu et al. [144] proposed a multiple view sampling-based multi-section CNN model for nodule classification and malignancy estimation from CT scans. Their proposed model is computationally lighter than the widely used and relatively efficient 3D-CNNs. They used sample slices extracted at different orientations, with spherical sampling performing the best, and a pre-trained MobileNet network to build their system. On the classification task, the proposed model achieved a sensitivity of 96% and AUC of 98% on the LUNA2016 dataset. They measure the severity of malignancy by training the logistic regression model to estimate the class probability of malignancy. It achieved an accuracy of 93.79% on malignancy estimation. Since it is a lightweight model, the model can work on smaller devices such as smart-phones, tablets, etc.

Ozdemir et al. [145] proposed an efficient end-to-end CAD system by coupling nodule detection with malignancy ranking step. They called the nodule detection system as $CAD_{e}$ (detection) whose output is an input for malignancy ranking step, $CAD_{x}$ (diagnosis) step in the complete CAD system. Training and evaluation were performed on the LUNA16 and Kaggle Bowl datasets [146]. The proposed model includes model uncertainty in its decision, making it relatively trustworthy in a real-world application. The proposed $CAD_{e}$ system achieved a sensitivity of 96.5%+ and 19.7 false positives per patient without using false positive reduction techniques. The $CAD_{x}$ system also achieves an AUC of 98%. The combination of the two systems was further tuned to build the full CAD system.

Bansal et al. [147] proposed Deep3DSCan for lung cancer segmentation and classification. They used a deep 3D segmentation algorithm to extract a 3D volume of interest from CTs. A combination of features extracted using a deep fine-tuned residual network and morphological features were used to train the ResNet based model. Training and testing used the publicly available LUNA16 dataset. The proposed architecture achieved an accuracy of 88% in segmentation and classification tasks with an F1-score of 0.88.

#### 3.5.4. Summary

Many of the papers discussed here studied the detection and classification of lung nodules from the LDCT images. The end-to-end papers covered in these reviews ([142,145,147]) can help to build an effective CAD system to assist the radiologist.Bansal et al. [147] seems to work better than the other works reviewed here since its performance covers both the detection and classification tasks (see Table 29). We must also consider MTANN-based papers since they deliver a satisfactory result with smaller data sizes. Papers reviewed here can be summarized in Table 30 and Table 31. Here we did not consider to create a table for domain expert approval since none was participated in the papers we reviewed.

#### 3.5.5. Deep Learning for Other Cancer Detection and Segmentation

In this sub-section, we included the application of deep learning on skin, liver and stomach cancer detection. Melanoma is skin cancer which is the deadliest cancer in its nature. In a normal diagnosis distinguishing melanoma lesions from nonmelanoma, lesions are very challenging [148]. For such difficulties, researchers have introduced an automatic skin cancer diagnosis system for detection and segmentation. As a result of uneasy visual characteristics of skin lesion that include inhomogeneous features and fuzzy boundaries. To overcome the challenges, Adegun and Viriri [148] proposed a deep learning-based automatic melanoma lesion detection and segmentation algorithm. They evaluated their approach in terms of Dice coefficient and accuracy 92% and 95% on ISIC 2017 dataset and accuracy and Dice coefficient of 95% and 93% on PH2 datasets.

Another deadly cancer with high morbidity is liver cancer. There are no widely recommended methods for early detection of liver cancer. For patients at higher risk, an imaging test such as CT, MRI, ultrasound and angiography can be used to test for liver cancer. If the physician believes in the need, a biopsy may be used to confirm the diagnosis. Hence, deep learning methods have been proposed in assisting physicians in the diagnosis of liver cancers, including hepatocellular carcinoma (HCC), liver metastasis, cysts, hemangioma and other masses [149]. A custom CNN for classifying HCC in liver cancer from MRI images, which achieved 92% sensitivity (Sn), 98% specificity (Sp) and 92% accuracy was proposed in [150]. In [151], VGGNet was used to develop a CAD system that identifies four types of liver cancers, cysts, hemangiomas, HCC and metastatic liver cancer from ultrasound images. The developed CAD system achieved an average accuracy of 88%. A hybridized fully convolutional neural network (HFCNN) was applied in the detection of liver cancer from abdominal CT images in [64]. HFCNN accurately segments 94.7% of the tumor volume.

Stomach (gastric) cancer is also another common cancer with the fourth-highest incidence rate and the third-highest mortality rate in the world [112]. The most common screening methods for stomach cancer are UGI series, serum pepsinogen (PG) testing, Hpylori serology and endoscopy [152]. Endoscopy is the most accurate of these methods, but it is highly invasive [152]. Some popular deep learning architectures, the inception, ResNet and VGGNet that were pre-trained on ImageNet were applied using transfer learning methods has been applied to identify benign ulcer and cancer from gastrointestinal endoscopic color images in [153]. The ResNet model achieved the highest performance with normal versus abnormal accuracies of 90% and ulcer versus cancer accuracy of 77%. A pre-trained Inception-Resnet-v2 model was trained and compared with endoscopists in classifying classification of gastric neoplasms in [154]. The Inception-Resnet-v2 model performance was lower in five-class (advanced gastric cancer, early gastric cancer, high-grade dysplasia, low-grade dysplasia and no neoplasm) classification, i.e., 76.4% vs. 87.6%. The Inception-Resnet-v2 model performance is comparable with endoscopist in the differentiation of gastric cancer and neoplasm (accuracy 76.0% vs. 82.0%).

## 4. Conclusions

The paper review indicates that deep learning methods have achieved state-of-the-art breast cancer, cervical cancer, brain tumor. colon cancer and lung cancer detection, feature extraction, classification and segmentation. In this article, a good number of deep learning-based methods for breast cancer, cervical cancer, brain tumor and colon cancer image analysis are studied. The developed and implemented methods employed a CNN approach combined with other techniques to mitigate the existing challenge arising from a large dataset demand for such systems to learn. Problems related to noise and corrupted images have been properly addressed in some of the works as they suggest utilizing normal images and limited dataset size in modes that encompass a combination of two or more architectures to discern breast, cervical, brain and colon abnormalities.

The use of enhanced activation functions have also been recommended to improve the overall performance of deep learning-based systems in medical image analysis. Moreover, many researchers have added multiple layers in the CNN architecture to increase the performance of the system. Some researchers worked on designing unique architectures for specific task properties instead of using CNN as it is. Subsequently, most of the methods are proved to produce a successful performance in terms of specificity, sensitivity and Dice score when tested on renowned datasets and BRATS challenges. The lack of sufficient datasets can be considered a challenge for many of the deep learning-based researches. All of the reviewed papers have not used different medical images other than MRI for brain and in most cases mammograms for breast.

## Figures and Tables

**Table 1:** World Health Organization 2018 statistical report through the global cancer observatory.

Cancer Type	New Cases (%)	Death Rate (%)
Breast Cancer	11.6	6.6
Colon Cancer	10.2	9.2
Brain Tumor	3.5	2.8
Cervical Cancer	3.2	2.5
Stomach Cancer	5.7	8.2
Liver Cancer	4.7	8.2
Lung Cancer	11.6	18.4

**Table 2:** Image datasets for breast cancer image analysis.

Dataset	Size	#Classes/Targets	Format	Type	Author/Repository, Year
MIAS	322	2	pgm	Mammography	Suckling, J. et al. [14]
DDSM	55,890		npy	Mammography	Scuccimarra [15]
InBreast		410	XML	Mammography	Moreira et al. [16]
Breast Cancer Wisconsin	568	3	csv	Mammography	Dua, D. and Graff, C. [17]
BreakHis	7909	2	png	Histology	Bukun [18]
BACH/ICIAR2018	400	4	tiff	Histology	G.Aresta [19]

**Table 3:** Summary of scientific papers on detection of breast cancer using diagnostic medical imaging techniques.

**Author and Citation**	**Dataset**	**AUC**	**Sn (%)**	**Sp (%)**	**Acc (%)**	**Target**
	Siemens and Hologic	0.933	-	-	-	Detection
Wu et al. [20]	Personal	0.895	-	-	-	Classification/Prediction
Shen et al. [2]	DDSM	0.88	-	-	-	Detection
(Single-Model)	INbreast	0.95	-	-	-	Detection
Shen et al. [2]	DDSM	0.91	86.1	80.1	-	Detection
(Four-Models Average)	INbreast	0.98	86.7	96.1	-	Detection
Zhu et al. [22] (Transfer learning)	-	0.53	-	-	-	Prediction
Zhu et al. [22] (SVM)	-	0.7	-	-	-	Prediction
Li et al. [23]	-	0.95	83	93.84	92.13	Classification
Zeiser et al. [24]	DDSM	0.86	92.32	80.47	85.95	Segmentation
Zhang et al. [28]	-	-	-	-	97.5	Detection
Zhou et al. [26]	-	0.86	90.8	69.3	83.7	Classification

**Table 4:** Summary of scientific papers on classification of breast cancer using histopathological images.

**Author and Citation**	**Dataset**	**Acc**	**Sn (%)**	**Sp (%)**
	Siemens and Hologic	0.933	-	-
Vang et al. [33]	ICIAR2018	87.5	-	-
	(H & E)			
Sharma and Mehra [32]	BreakHis	93.97	-	-
Sheikh et al. [29]	ICIAR2018	83	-	-
	and BreakHis	98		
Li et al. [30]	ICIAR2018	88	-	-
Yan et al. [31]	ICIAR2018	91	-	-
Alzubaidi et al. [21]	ICIAR 2018	97.4	-	

**Table 5:** Summary of breast cancer scientific papers in terms of convolutional neurla network (CNN) architecture and type of environment used in the selected papers.

Authors	Network	Pre-Training	Transfer Learning	Environment
Wu et al. [20]	ResNet-22	Yes	No	TensorFlow
Shen et al. [2]	ResNet-50, VGGNet-16	Yes	Yes	-
Vang et al. [33]	Inception V3	Yes	No	TensorFlow
Zhu et al. [22]	GoogleNet	Yes	Yes	Caffe
Li et al. [23]	VGGNet-16	Yes	Yes	-
Sharma and Mehra [32]	VGGNet-16, VGGNet-19, ResNet50	Yes	Yes	Keras, TensorFlow
Zeiser et al. [24]	U-net	No	No	-
Zhang et al. [28]	U-net	No	No	TensorFlow
Zhou et al. [26]	3D DensNet	No	No	-
Sheikh et al. [29]	MSI-MFNet	No	No	Keras
Li et al. [30]	IDSNet	Yes	Yes	Tensorflow
Yan et al. [31]	Inception-V3	Yes	Yes	Tensorflow
Alzubaidi et al. [21]	ResNet	Yes	Yes	-

**Table 6:** Summary of breast cancer scientific papers in terms of publication year, name of journal/conference for the selected papers and its impact factor with year of impact factor.

Authors	Publication Year	Journal/Conf.	Impact Factor	Year of Impact Factor
Wu et al. [20]	2020	ITMI	6.85	2020
Shen et al. [2]	2019	Scientific Reports	3.998	2019
Vang et al. [33]	2018	CBM	5.4	2019
Zhu et al. [22]	2019	CBM	3.434	2020
Li et al. [23]	2019	European Radiology	4.101	2019
Sharma and Mehra [32]	2020	Journal of Digital Imaging	2.99	2018
Zeiser et al. [24]	2020	Journal of Digital Imaging	2.99	2018
Zhang et al. [28]	2018	Academic Radiology	2.50	2020
Dembrower et al. [34]	2020	Radiology	7.608	2018
Zhou et al. [26]	2019	Journal of Magnetic Resonance Imaging	2.112	2018
Sheikh et al. [29]	2020	MDPI, Cancers	6.126	2019
Li et al. [30]	2020	Plos One	2.74	2019
Yan et al. [31]	2020	Elsevier, Methods	3.812	2019
Alzubaidi et al. [21]	2020	MDPI, electronics	2.412	2019

**Table 7:** Summary of breast cancer scientific papers in terms of comparison to specialists and/or traditional techniques.

Author and Citation	Comparison to Specialists	Comparison to Traditional Technique (Yes/No)
Hagos et al. [35]	No	No
Wu et al. [20]	Yes	No
Shen et al. [2]	No	No
Vang et al. [33]	No	No
Zhu et al. [22]	No	No
Li et al. [23]	No	No
Sharma and Mehra [32]	No	Yes
Zeiser et al. [24]	No	Yes
Zhang et al. [28]	No	No
Zhou et al. [26]	Yes	Yes
Sheikh et al. [29]	No	Yes
Li et al. [30]	No	Yes
Yan et al. [31]	No	Yes
Alzubaidi et al. [21]	No	yes

**Table 8:** Image datasets for cervical cancer.

Dataset	Size	#Classes/Targets	Format	Type	Author, Year
Herlev	917	187	Bit Map(BMP)	Histology	Dr J. Jantzen [43]
DANS-KNAW	963	4	jpg	Histology	Hussien [44]
CRIC	400	6	png and csv	Histology	M.T. Rezende et al. [45]
Zenodo	962	4	jpg	Histology	Franco et al. [46]
ALTS	938	2	jpg	Colposcopy	Alts Group [47]
MobileODT	1448	3	jpg	Colposcopy	MobileODT [48]

**Table 9:** Summary of selected papers on nuclei segmentation.

Authors	Method	Dataset	Acc	P	R	F1	Sp	Sn	ZSI
Zhao et al. [50]	Progressive Growing	Herlev	0.925	0.901	0.968				0.925
	of U-net+(PGU-net+)								
Liu et al. [53]	Mask-RCNN with LFCCRF	Herlev		0.96	0.96				0.95
Sompawong et al. [52]	Mask-RCNN	TU	89.8%				94.3%	72.5%	

**Table 10:** Summary of selected papers on cervical cell segmentation.

Authors	Method	Dataset	Acc	P	R	ZSI	DSC
Kurnianingsih et al. [54]	Mask R-CNN	Herlev		0.92	0.91	0.91	
Song et al. [49]	CNN with Shape information	Herlev					0.92
Liang et al. [42]	comparison based Faster R-CNN	local		26.3	35.7		

**Table 11:** Summary of selected papers on cervical cell binary classification.

Authors	Method	Dataset	Acc(%)	Sn(%)	Sp(%)	AUC	F1	P	R
Zhang et al. [55]	DeepPap	Herlev	98.3	-	98.3	0.99	-	-	-
Hyeon et al. [56]	VGG16	SVM	local	-	-	-	0.78	0.78	0.78
Lin et al. [57]	GoogleNet5C	Herlev	94.5	-	-	-	-	-	-
Chen et al. [60]	Mask R-CNN 7 class	local-	87.4	88.6	86.1	-	-	-	-
Kurnianingsih et al. [54]	Mask R-CNN	Herlev	98.1	96.7	98.6	96.5	-	-	-
Promworn et al. [61]	densenet161	Herlev	94.38	100	-	-	-	-	-
Yutao Ma et al. [62]	CNN and SVM	OCM image	-	86.7	93.5	0.96	-	-	-
Ahmed et al. [63]	CaffNet+ELM	Herlev	99.5	-	-	-	-	-	-
Dong et al. [64]	Inception-V3	Herlev	98.23	99.4	96.7	-	-	-	-
Martinez-Mias et al. [65]	CaffeNet	Local	88.8	92	83	-	-	-	-

**Table 12:** Summary of selected papers on cervical cell multiclass classification.

Authors	Method	Dataset	Acc	Sn	Sp	Others
	regressor 7 classes					
Yutao Ma et al. [62]	CNN and SVM 5 classes	OCM image	88.3			
Lin et al. [57]	GoogleNet5C 4 classes	Herlev Dataset	71.3			
Lin et al. [57]	GoogleNet5C seven classes	Herlev Dataset	64.5			
Kurnianingsih et al. [54]	Mask R-CNN 7 class	Herlev	95.9	96.2	99.3
Promworn et al. [61]	densenet161 7 classes	Herlev dataset	68.54	68.18	69.57	
Ahmed et al. [63]	CaffNet+ELM	Herlev	91.2	-	-	-
Martinez-Mias et al. [65]	CaffeNet	Local	55.6	-	-	-
Xiang et al. [66]	YOLOv3+InceptionV3	local	89.3	97.5	67.8	-

**Table 13:** Summary of cervical cancer scientific papers in terms of CNN architecture, and type of environment used in the selected papers.

Author and Citation	Network	Pre-Training	Transfer Learning	Environment
Zhao et al. [50]	U-Net	No	No	-
Liu Y. et al. [53]	Mask-RCNN	Yes	No	Tensorflow
Sompawong et al. [52]	Mask-RCNN	Yes	Yes	-
Kurnianingsih et al. [54]	Mask-RCNN and VGGNet	Yes	Yes	-
Song et al. [49]	CNN-Custom	No	No	-
Lianget al. [42]	ResNet50	Yes	Yes	Tensorflow
Zhang et al. [55]	ConvNet	Yes	Yes	Caffe
Hyeon et al. [56]	CNN	Yes	Yes	-
Yutao Ma et al. [62]	VGG-16	Yes	Yes	Tensorflow
Lin et al. [57]	GoogLeNet	Yes	Yes	Caffe
Promworn et al. [61]	DenseNet161	No	No	PytTorch
Wimpy and Suyanto [68]	Capsule Network	Yes	No	Tensorflow
Gorantla et al. [69]	ResNet101	yes	Yes	-
Arora et al. [70]	CNN-Custom	No	No	-
Ahmed et al. [63]	CaffeNet	yes	yes	Caffe
Martinez-Mias et al. [65]	CaffeNet	yes	yes	Caffe

**Table 14:** Summary of cervical cancer scientific papers in terms of article publication year, name of the journal for the selected papers and its impact factor with year of impact factor.

Author and Citation	Publication Year	Journal/Conference	Impact Factor	Impact Assigned Year
Zhao et al. [50]	2019	MMMI 2019	-	-
Liu Y. et al. [53]	2018	IEEE Access	4.098	2018
Sompawong et al. [52]	2019	Conference ACEMBS	0.54	2019
Kurnianingsih et al. [54]	2019	IEEE Access	4.098	2018
Song et al. [49]	2016	Conference ISBI	1.51	2019
Liang et al. [42]	2019	Neurocomputing	3.317	2016
Zhang et al. [55]	2017	JBHI	5.223	2020
Hyeon etal. [56]	2017	Conference ICMDM	-	-
Yutao Ma et al. [62]	2019	IEEE Transaction on Biomedical Engineering	4.78	2019
Lin et al. [57]	2019	IEEE Access	4.098	2018
Promworn et al. [61]	2019	Conference ICNEMS	0.312	2019
Wimpy and S. Suyanto [68]	2019	Conference ISRITI	-	-
Gorantla et al. [69]	2019	BIBE	0.392	2012
Arora et al. [70]	2018	Conference ICSCCC	0.91	2019
Ahmed et al. [63]	2019	Future Generation computer systems	6.125	2019
Dong et al. [64]	2020	ASCJ	5.5	2020
Martinez-Mias et al. [65]	2020	ESWA	5.45	2020

**Table 15:** Summary of cervical cancer scientific papers in terms of comparison to specialists and/or traditional techniques.

Author and Citation	Comparison to Specialists	Comparison to Traditional Technique (Yes/No)
Zhao et al. [50]	No	Yes
Liu Y. et al. [53]	No	Yes
Sompawong et al. [52]	No	Yes
Kurnianingsih et al. [54]	No	Yes
Song et al. [49]	No	Yes
Lianget al. [42]	No	Yes
Zhang et al. [55]	Yes	No
Hyeon et al. [56]	No	No
Yutao Ma et al. [62]	Yes	No
Lin et al. [57]	No	Yes
Promworn et al. [61]	No	Yes
Wimpy and S. Suyanto [68]	No	Yes
Gorantla et al. [69]	No	Yes
Arora et al. [70]	No	No
Ahmed et al. [63]	No	Yes
Dong et al. [64]	No	Yes
Martinez-Mias et al. [65]	No	No

**Table 16:** Image datasets for brain tumor (N—normal, AB—abnormal).

Dataset	Size	#Classes	Format/Targets	Type	Author, Year
LBPA40	288	2	html	MRI	Shattuck et al. [85]
BRATS 2015	43,708	2	.mha	MRI	Menze et al. [86]
BRATS2013	1799	2	.mha	MRI	Menze et al. [86]
RIDER_NEURO_MRI	29	2	.tcia	MRI	Barboriak et al. [87]
SUH	49	2	-	MRI	Fabelo et al. [88]
HMS	66	2	.gif	MRI	Keith A. Johnson
FBT	3064	2	.mat	MRI	C. Jun [89]
NHTM	3064	2	.png	MRI	C. Jun [89]
GCE	150	2	.png	MRI	Jun Cheng [90]

**Table 17:** Summary of selected papers on brain tumor segmentation.

Authors	Method Learning	Dataset	Acc.	P	R	F	Sp	Sn	Dice	PPV
Alkassar et al. [91]	DNN+FCN+VGG-16	BRATS2015	0.98						0.89	
Amiri et al. [92]	RF-SVM	BRATS							0.72	
Chahal et al. [93]	CNN	BRATS2013		0.96				0.93	0.95	
Ding et al. [94]	RDM-Net	BRATS2015							0.86	
Mallick et al. [95]	DWA-DNN	RIDER_NEURO_MRI	0.93			0.93	0.92	0.94		
Ramirez et al. [96]	CNN+TVS	Flair-MRI Brats2015		0.84	0.88				0.86	
Sajid et al.[97]	hybrid CNN	BRATS 2013					0.91	0.86	0.86	
Wang et al. [98]	WRN-PPNet	BRATS2015						0.92	0.94	0.97
Zhao et al. [99]	FCNNs and CRF-RNN	BRATS 2013–16						0.82	0.84	0.89
Kuzina et al. [100]	UNet-DWP	BRATS2018							0.76	
Zeineldin et al. [101]	DeepSeg	BRATS 2019							0.81–0.84	
Fabelo et al. [102]	HSI+2D-CNN	SUH	80					80–100		

**Table 18:** Summary of selected papers on brain tumor classification.

Authors	Method	Dataset	Acc.	P	R	F	Sp	Sn	MCC	G-Mean
Mohsen et al. [80]	DWT-DNN	Harvard		0.97	0.97	0.97				
Alqudah et al. [105]	CNN	Online	98.40	98.19			99.19	98.18		
Ucuzal et al. [106]	CNN Multiclass	NHTM	99.74	99.58		99.59	99.81	99.60	99.39	99.70
Selvy et al. [109]	PNN	GCE	90			100	85.75			
Sultan et al. [110]	CNN	NHTM	96.13–98.7							
Badža and Barjaktarovic [111]	CNN	NHTM	96.56							

**Table 19:** Summary of brain tumor scientific papers in terms of article publication year, name of journal for the selected papers and its impact factor with year of impact factor.

Author and Citation	Publication Year	Journal	Impact Factor	Impact Assigned Year
Alkassar et al. [91]	2019	ICECCPCE19 Conference	0.627	2019
Amiri et al. [92]	2016	ATSIP 2016 Conference	0.17	2019
Chahal et al. [93]	2019	RDCAPE Conference	-	-
Ding et al. [94]	2019	IEEE Access	3.745	2019
Mallick et al. [95]	2019	IEEE Access	3.745	2019
Ramirez et al. [96]	2018	ISBI Conference	1.51	2019
Sajid et al.[97]	2019	Arabian Journal for Science and Engineering	0.33	2019
Wang et al. [98]	2019	IJCNN Conference	0.37	2019
Zhao et al. [99]	2018	Medical Image Analysis	3.88	2019
Kuzina et al. [100]	2019	Frontiers in Neuroscince	3.7	2020
Mohsen et al. [80]	2018	Future Computing and Informatics	3.88	2019
Alqudah et al. [105]	2019	IJATCSE	0.2	2019
Ucuzal et al. [106]	2019	ISMSIT	0.84	2019
Zeineldin et al. [101]	2020	IJCARS	1.961	2017
Fabelo et al. [102]	2019	MDPI	3.275	2019
Selvy et al. [109]	2019	IJSRCSEIT	1.638	2016
Sultan et al. [110]	2019	IEEE Access	3.745	2019
Badža and Barjaktarovic [111]	2020	MDPI	2.474	2019

**Table 20:** Summary of brain tumor scientific papers in terms of comparison to specialists and/or traditional techniques.

Author and Citation	Comparison to Specialists (Yes/No)	Comparison to Traditional Technique (Yes/No)
Alkassar et al. [91]	Yes	Yes
Amiri et al. [92]	No	Yes
Chahal et al. [93]	No	Yes
Ding et al. [94]	No	Yes
Mallick et al. [95]	No	Yes
Ramirez et al. [96]	Yes	Yes
Sajid et al.[97]	No	Yes
Wang et al. [98]	Yes	Yes
Zhao et al. [99]	Yes	Yes
Kuzina et al. [100]	No	Yes
Mohsen et al. [80]	No	Yes
Alqudah et al. [105]	No	Yes
Ucuzal et al. [106]	Yes	No
Zeineldin et al. [101]	Yes	Yes
Fabelo et al. [102]	Yes	Yes
Selvy et al. [109]	No	No
Sultan et al. [110]	Yes	Yes
Badža and Barjaktarovic [111]	No	Yes

**Table 21:** Summary of brain tumor scientific papers in terms of CNN architecture, and type of environment used in the selected papers.

Author and Citation	Network	Pre-Training	Transfer Learning	Environment
Alkassar et al. [91]	VGGNet-16	Yes	Yes	
Amiri et al. [92]	RF+SVM	Yes	Yes	
Chahal et al. [93]	CNN	Yes	Yes	
Ding et al. [94]	RDM-Net	Yes	Yes	
Mallick et al. [95]	DWA-DNN	Yes	Yes	Tensor flow
Ramirez et al. [96]	CNN+TVS	Yes	Yes	Tensor flow
Sajid et al.[97]	hybrid CNN	Yes	Yes	Tensor Flow
Wang et al. [98]	WRN-PPNet	Yes	Yes	Tensor flow
Zhao et al. [99]	FCNNs and CRF-RNN	Yes	Yes	Tensor flow
Kuzina et al. [100]	UNet-DWP	Yes	Yes	
Mohsen et al. [80]	DWT-DNN	Yes	Yes	
Alqudah et al. [105]	VGGNet-19	Yes	Yes	
Ucuzal et al. [106]	UNet-DWP	Yes	Yes	Tensor flow and Keras
Zeineldin et al. [101]	ResNet+DenseNet+NasNet	Yes	Yes	Keras, Tensor Flow
Fabelo et al. [102]	UNet	Yes	Yes	Tensor Flow
Selvy et al. [109]	GLCM+PNN	Yes	Yes	
Sultan et al. [110]	CNN	Yes	Yes	Matlab 2018b and Python
Badža and Barjaktarovic [111]	CNN	Yes	Yes	Matlab 2018b

**Table 22:** Publicly available datasets for colorectal cancer (CRC), UMCM—University Medical Center Mannheim, CVC—computer vision center.

Dataset	Size	#Classes/Targets	Format	Type	Author, Year
CVC-EndoSceneStill	912	4	bmp	Colonoscopy	Vázquez et al. [116], 2017
CVC-ColonDB	300	4	bmp	Colonoscopy	J. Bernal et al. [117], 2012
CVC-ClinicDB	612	4	tiff	Colonoscopy	J. Bernal et al. [118], 2015
UMCM	500	8	mat	histology	Kather et al. [119], 2016

**Table 23:** Summary of selected papers on detection and classification for colorectal cancer histological slides.

Authors	Method	Dataset	Acc	P	R	F1	DSC	H
Kainz et al. [120]	Separator-Net and Object-Net	MICCAI2015	96	59	74	62	-	-
Graham et al. [113]	MILD-Net	$GlaS^{+}$	-	-	-	87	88	142
Chamanzar et al. [122]	WSMTL	local	93	-	-	79.1	78.4	-
Sari et al. [123]	DeepFeature	local	-	82.3	89.9	85.1	-	-
Shapcott et al. [125]	CNNs	local and TCGA	65	-	-	-	-	-
Sirinukunwattana et al. [121]	SC-CNN+NEP & s-CNN	MICCAI2015	-	-	-	-	69	-
Tang et al. [126]	Segnet	MICCAI2015	-	-	-	-	87.2	104.61
Vuong et al. [127]	Multitask DensNet121	local	85.1	-	-	-	-	-
Sabol et al. [128]	CFSCMC	UMCM	92.78	-	-	-	-	-

**Table 24:** Summary of selected papers on colorectal cancer polyp detection.

Authors	Method	Dataset	Acc	P	R	F1	Sp	Sn	PPV
Ornela Bardhi et al. [130]	SegNet	EITs	96.7	-	-	-	-	-	-
Bour et al. [131]	Resnet50	local	87.1	87.1	87.1	87.1	93	-	-
Liu et al. [132]	faster_rcnn_inception_resnet_v2	local	90.6	-	-	-	-	-	-
Ozawa et al. [133]	SSD	local	-	-	-	-	-	92	86
Nadimi et al. [134]	mZF-net+ResNet	local	98	-	-	-	98.1	96	-

**Table 25:** Summary of colorectal cancer in terms of article publication year, name of journal for the selected papers and its impact factor with year impact factor has been assigned.

Author and Citation	Publication Year	Journal/Conference	Impact Factor	Impact Assigned Year
Kainz et al. [120]	2017	PeerJ	2.38	2019
Graham et al. [113]	2018	Medical Image Analysis	8.79	2018
Chamanzar et al. [122]	2020	ISBI conference	2.283	2019
Sari et al. [123]	2019	IEEE Transactions on Medical Imaging	9.71	2019
Shapcott et al. [125]	2019	Frontiers in bioengineering and biotechnology	3.644	2020
Sirinukunwattana et al. [121]	2016	IEEE transactions on medical imaging	9.71	2019
Tang et al. [126]	2018	Conference YAC	1.461	2019
Vuong et al. [127]	2020	Conference ICEIC	0.76	2019
Ornela Bardhi et al. [130]	2017	Conference ISSPIT	1.393	2019
Bour et al. [131]	2017	Conference ISSPIT	1.393	2019
Liu et al. [132]	2019	Conference ISNE	0.152	2019
Ozawa et al. [133]	2020	Therapeutic advances in gastroenterology	4.08	2020
Nadimi et al. [134]	2020	CEE	2.663	2020
Sabol et al. [128]	2020	YJBIN	3.526	2020

**Table 26:** Summary of colorectal cancer in terms of CNN architecture and type of environment used in the selected papers.

Author and Citation	Network	Pre-Training	Transfer Learning	Environment
Kainz et al. [120]	Object-Net and SeparatorNet—custom	No	No	Matlab
Graham et al. [113]	MILD-Net—custom	No	No	Tensorflow
Chamanzar et al. [122]	U-net and Resnet	Yes	Yes	PyTorch
Sari et al. [123]	DeepBelief	Yes	Yes	-
Shapcott et al. [125]	-	No	No	Tensorflow
Sirinukunwattana et al. [121]	-	No	No	Matlab
Tang et al. [126]	SegNet	No	No	Caffe
Vuong et al. [127]	DensNet121	No	No	PyTorch
Ornela Bardhi et al. [130]	SegNet	No	No	Tensorflow
Bour et al. [131]	ResNet50	Yes	Yes	Tensorflow
Liu et al. [132]	faster_rcnn_inception_resnet_v2	No	No	Tensorflow
Ozawa et al. [133]	Single Shot MultiBox Detector (SSD)	No	No	Caffe
Nadimi et al. [134]	mZF-Net+ResNet	Yes	Yes	Matlab 2018a
Sabol et al. [128]	Xception+CFCMC	Yes	Yes	-

**Table 27:** Summary of colorectal cancer papers in terms of comparison to specialists and/or traditional techniques.

Author and Citation	Comparison to Specialists	Comparison to Traditional Technique (Yes/No)
Kainz et al. [120]	No	No
Graham et al. [113]	No	Yes
Chamanzar et al. [122]	No	Yes
Sari et al. [123]	No	No
Shapcott et al. [125]	No	No
Sirinukunwattana et al. [121]	No	Yes
Tang et al. [126]	No	Yes
Vuong et al. [127]	No	No
Ornela Bardhi et al. [130]	No	No
Bour et al. [131]	Yes (Approval)	No
Liu et al. [132]	No	Yes
Ozawa et al. [133]	No	No
Nadimi et al. [134]	No	No
Sabol et al. [128]	Yes	Yes

**Table 28:** Publicly available datasets for CRC, UMCM—University Medical Center Mannheim, CVC—computer vision center.

Dataset	Size	#Classes/Targets	Format	Type	Author, Year
UCI ML repository	32	3		CSV	Hong and Yang [137], 1991
SPIE-AAPM-NCI	22489	2	dicom	CT	Armato et al. [138], 2015
Lung Nodule Malignancy	6690	2	hdf5	CT	Scott Mader [139], 2017
LUNA2016	888	2	mhd.zip	CT	Consortium for Open Medical Image Computing [119], 2016

**Table 29:** Summary of selected papers on lung cancer detection and classification.

Authors	Method	Dataset	Acc	FPR	Sp	Sn	AUC
Tajbakhsh and Suzuki [142]	MTANN, detection	local	-	2.7	-	100	
Tajbakhsh and Suzuki [142]	MTANN, classification	local	-	-	-	-	0.88
Gu et al. [143]	3D-CNN, detection	LUNA16	-	2.5	-	90	
Sahu et al. [144]	multi-section MobileNet	LUNA16	93.8	-	-	96	0.98
Ozdemir et al. [145]	V-Net, classification	LUNA16	-	19	-	96.5	0.98
Bansal et al. [147]	ResNet	LUNA16	88	-	89.7	87	0.88

**Table 30:** Summary of lung cancer in terms of article publication year, name of journal for the selected papers and its impact factor with the year impact factor has been assigned.

Author and Citation	Publication Year	Journal/Conference	Impact Factor	Impact Assigned Year
Tajbakhsh and Suzuki [142]	2017	Pattern Recognition	7.196	2019
Gu et al. [143]	2018	CBM	3.43	2019
Sahu et al. [144]	2019	IEEE-JBHI	5.180	2020
Ozdemir et al. [145]	2020	IEEE Transactions on Medical Imaging	9.71	2020
Bansal et al. [147]	2020	IET Image Processing	2.61	2020

**Table 31:** Summary of Colorectal cancer in terms of CNN architecture and type of environment used in the selected papers.

Author and Citation	Network	Pre-Training	Transfer Learning	Environment
Tajbakhsh and Suzuki [142]	MTANN	-	-	Caffe
Gu et al. [143]	3D-CNN	-	-	Keras
Sahu et al. [144]	Mobile-Net	Yes	Yes	Keras
Ozdemir et al. [145]	Vnet	-	-	-
Bansal et al. [147]	Resnet	-	-	Pythorch
